# Glycogen synthase kinase 3 inhibition controls *Mycobacterium tuberculosis* infection

**DOI:** 10.1016/j.isci.2024.110555

**Published:** 2024-07-20

**Authors:** Sandra Peña-Díaz, Joseph D. Chao, Celine Rens, Hasti Haghdadi, Xingji Zheng, Keegan Flanagan, Mary Ko, Tirosh Shapira, Adrian Richter, Danay Maestre-Batlle, Julio Ortiz Canseco, Maximiliano Gabriel Gutierrez, Khanh Dao Duc, Steven Pelech, Yossef Av-Gay

**Affiliations:** 1 Department of Microbiology and Immunology, Life Sciences Institute, University of British Columbia, Vancouver, BC, Canada; 2 Department of Medicine, University of British Columbia, Vancouver, BC, Canada; 3 Institut für Pharmazie, Martin-Luther-Universität Halle-Wittenberg, Halle (Saale), Germany; 4 Host-pathogen Interactions in Tuberculosis Laboratory, The Francis Crick Institute, London, UK; 5 Department of Mathematics, University of British Columbia, Vancouver, BC, Canada; 6 Kinexus Bioinformatics Corporation, 8755 Ash Street, Vancouver, BC, Canada

**Keywords:** Molecular biology, Microbiology, Medical Microbiology

## Abstract

Compounds targeting host control of infectious diseases provide an attractive alternative to antimicrobials. A phenotypic screen of a kinase library identified compounds targeting glycogen synthase kinase 3 as potent inhibitors of *Mycobacterium tuberculosis* (Mtb) intracellular growth in the human THP-1 cell line and primary human monocytes-derived macrophages (hMDM). CRISPR knockouts and siRNA silencing showed that GSK3 isoforms are needed for the growth of Mtb and that a selected compound, P-4423632 targets GSK3β. GSK3 inhibition was associated with macrophage apoptosis governed by the Mtb secreted protein tyrosine phosphatase A (PtpA). Phospho-proteome analysis of macrophages response to infection revealed a wide array of host signaling and apoptosis pathways controlled by GSK3 and targeted by P-4423632. P-4423632 was additionally found to be active against other intracellular pathogens. Our findings strengthen the notion that targeting host signaling to promote the infected cell’s innate antimicrobial capacity is a feasible and attractive host-directed therapy approach.

## Highlights

• GSK3 inhibitors control Mtb growth inside macrophages
• CRISPR knockout and RNAi of GSK3 control Mtb growth inside macrophages
• Signaling inhibitors of GSK3 provide a host-directed alternative to antibiotics

## Graphical abstract

**Figure:**
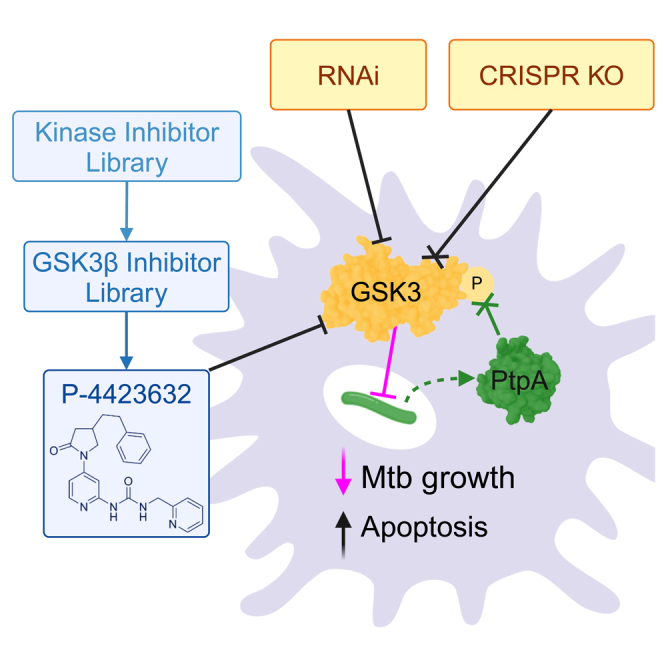

## Introduction

Tuberculosis (TB), caused by *Mycobacterium tuberculosis* (Mtb), is the major cause of mortality worldwide from a single infectious agent. Although the estimated latent infection rate may reach 25–33% of the world’s population,^1^ on average, only 5–10% of those infected will develop active TB disease over their lifetime^2^ indicating a key role of host determinants in dictating infection outcome.

The innate response provides the first line of defense against infection by Mtb, and postulates a promising avenue for host-directed therapies (HDTs), which are gaining attention as an alternative approach to fight this notorious pathogen. HDTs are defined as small molecules that target host pathways, enabling the host to either increase its antimicrobial capacity or reduce inflammation.^3^ Examples of such small molecules identified through drug repurposing are the nonsteroidal anti-inflammatory drug ibuprofen, and the protein-tyrosine kinase inhibitor imatinib.^4^ HDTs have been proposed for improving TB treatment outcomes and reducing the duration of therapy. Since HDTs do not target Mtb but rather assist the host in fighting infection, they are hypothesized to have reduced chances of generating antimicrobial resistance.^4^^,^^5^^,^^6^ Indeed, targeting autophagy, the cellular process that allows the orderly degradation and recycling of cellular components, as an HDT during the innate response was shown to be an effective approach for controlling Mtb.^7^^,^^8^

The macrophage constitutes the first line of host defense against invading microorganisms. However, Mtb has evolved multiple strategies to avoid being killed by the alveolar macrophage, allowing it to survive and replicate inside the host. One of these strategies is the secretion of protein effectors that disrupt the macrophage’s innate antimicrobial defenses, such as protein-tyrosine phosphatase A (PtpA).^9^^,^^10^ PtpA blocks phagosomal acidification and maturation by binding to subunit H of the macrophage V-ATPase complex and dephosphorylating VPS33B of the class C VPS macrophage complex.^9^^,^^10^ Recent studies show that Mtb modulates host intracellular signaling^11^^,^^12^ and that PtpA has a broader global effect on host cell signaling proteins.^9^^,^^10^^,^^11^ This, together with the promising properties of host kinase inhibitors as modulators of Mtb intracellular growth,^12^^,^^13^ merits screening libraries of signaling inhibitors to identify associated cellular pathways that can be exploited as HDTs in the fight against TB.

## Results

### GSK3 inhibitors restrict Mtb growth in a macrophage infection model

**Figure 1:**
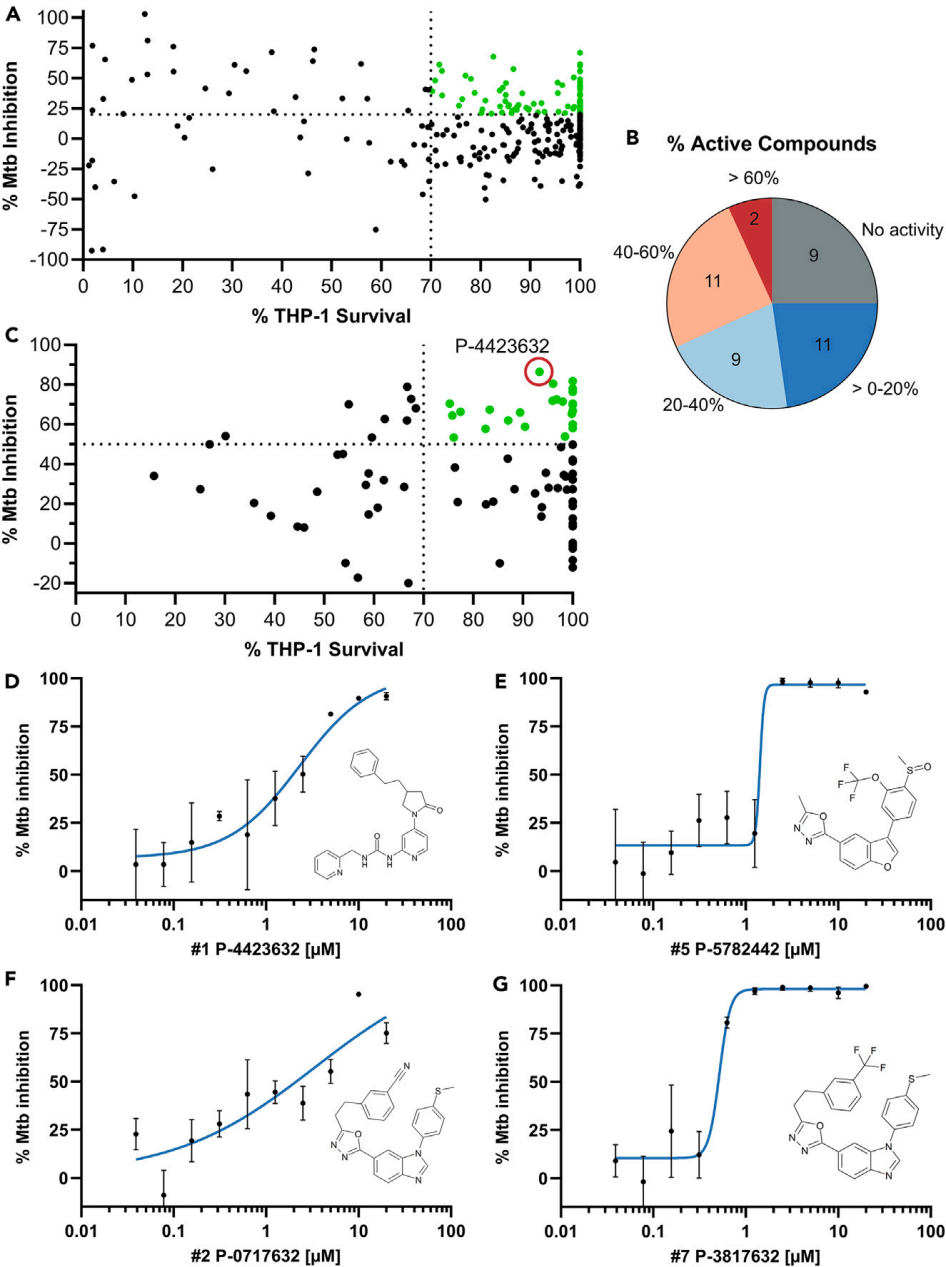
Intracellular screening of kinase inhibitor libraries identified GSK3β as a key host controller of Mtb infection (A) Screening of 313 unique compounds from the PKIS/UNC library at 10 μM concentration against THP-1 cells infected with RFP-expressing intracellular Mtb, using the CellInsight CX5 HCS platform (CX5). Z’ = 0.58 ± 0.12 across 8 plates. Mtb % inhibition, calculated based on the intracellular fluorescence intensity dual normalized to the BDQ positive control (100% inhibition) and the negative DMSO vehicle control (0% inhibition), was plotted as a function of THP-1 cell survival as measured by DAPI-stained nuclei cell counts compared to the negative control. Green circles in upper right quadrant represent compounds with greater than 20% Mtb inhibition and 70% THP-1 survival. (B) Distribution of GSK3β inhibitors based on their intracellular inhibition of Mtb. GSK3 inhibitors from the above library were rescreened against luciferase-expressing Mtb in THP-1 cells using a luciferase assay. Mtb % inhibition was calculated based on the relative luminescence, dual normalized to the Rifampicin positive control (100% inhibition) and the negative DMSO control (0% inhibition). Over half of the tested compounds (22/42) demonstrated at least 20% inhibition of Mtb intracellular growth. Z’ = 0.56. (C) Screening of a GSK3β focused library of compounds (Takeda) at 10 μM against luciferase-expressing Mtb in THP-1 macrophages using a luciferase assay. Mtb % inhibition (as calculated in B) was plotted as a function of THP-1 cell survival as determined by MTT assay. Green circles in upper right quadrant represent compounds with greater than 50% Mtb inhibition and 70% THP-1 survival; compound P-4423632 circled. (D–G) Dose-dependent inhibitory activity of four select GSK3 inhibitors against intracellular Mtb and the corresponding compound structures (inset). THP-1 cells infected with luciferase-expressing Mtb were treated with 2-fold serial dilutions of the indicated compounds. Mtb % inhibition was calculated as in (B). Non-linear regression (variable slope) was used to fit the data of the log (inhibitor) vs. response (±SD) using GraphPad Prism software; *N* = 3.

Following the rationale that modulation of host cell signaling can assist in controlling infection, we used a chemical biology approach, whereby specific inhibitors designed to target mammalian signaling proteins were screened for their ability to restrict intracellular Mtb growth. We screened a publicly available library of eukaryotic protein kinase inhibitors, the published kinase inhibitor set (PKIS),^13^ for their effect on Mtb growth in THP-1 macrophages. Multiple rounds of screening using high content screening (HCS) were conducted,^14^ and assay results, summarized in Figure 1A, identified 103 active compounds with at least 20% inhibition of Mtb intracellular growth out of 313 compounds screened. As our HCS assay monitors the numbers of both intracellular bacteria and host macrophages, we excluded any cytotoxic compounds causing a loss of more than 30% of host macrophages. Compounds targeting p38, GSK3, EGFR, TIE2, VEGRF2, and C-RAF had the highest numbers of identified hits per kinase target (Figure S1). While p38 MAPK had an additional hit compared to the 19 hits identified for GSK3, GSK3 targeting-compounds had the largest variety of chemotypes with at least one active compound identified for each chemotype. Based on these findings, and our previous published results identifying GSK3 as an important target for PtpA host modulation,^11^ we followed up our studies on compounds targeting GSK3. We performed another screen against 42 of the GSK3 inhibitors included within the PKIS library using a separate luciferase assay to verify our results. We obtained similar results to the HCS assay, identifying 22 compounds with at least 20% reduction in Mtb intracellular growth, 13 of which inhibited growth by at least 40% (Figure 1B).

GSK3 is a well-studied kinase,^15^ existing as two highly homologous isoforms, α and β, encoded by distinct genes. It is unique in function, constitutively active and generally inhibited in response to stimulation.^16^ Our HCS identified GSK3 as a viable target for HDT, however, the compounds we tested showed somewhat moderate effects. We therefore followed up with a screen of a focused library of GSK3β inhibitors, obtained from Takeda Pharmaceutical Company Ltd. which includes 88 highly selective 1,3,4-oxadiazole derivatives,^17^^,^^18^ with no detected activity against Mtb grown in broth under higher concentrations in an *in vitro* growth assay (Figure S2). We screened this GSK3β inhibitor library using luciferase assays combined with MTT assays,^14^^,^^19^ monitoring Mtb growth inside THP-1 human macrophages. As seen in Figure 1C, 32 out of the 88 GSK3 inhibitors reduced Mtb intracellular growth by 50% or higher in comparison to the DMSO control. An MTT cytotoxicity assay indicated that 68% of the inhibitors had an acceptable toxicity (>70% viable THP-1 cells). We selected the top four compounds from the Takeda GSK3β library, verified that they are active against GSK3β (Figure S3) and showed that they act in a dose-dependent manner against intracellular Mtb with minimal inhibitory concentrations (MIC50 or MIC90) below 10 μM (Figure 1D; Table S1). P-4423632, chosen as our hit compound for further studies, was inactive in a disk-diffusion antibiotics susceptibility assay against a panel of bacteria, including Mtb, grown *in vitro* in solid culture media (Table S2). P-4423632 was active against Mtb *in broth* with an MIC 50 of 38.8 μM, over 10-fold higher compared to its intracellular activity (Table S3). P-4423632 was also found to be active in a dose-dependent manner against the gram-negative intracellular bacterium *Campylobacter jejuni* in Caco-2 cells and *Salmonella enterica* serovar Typhimurium in THP-1 cells (Figure S4).

### GSK3 inhibition controls intracellular Mtb in infected THP-1 cells

**Figure 2:**
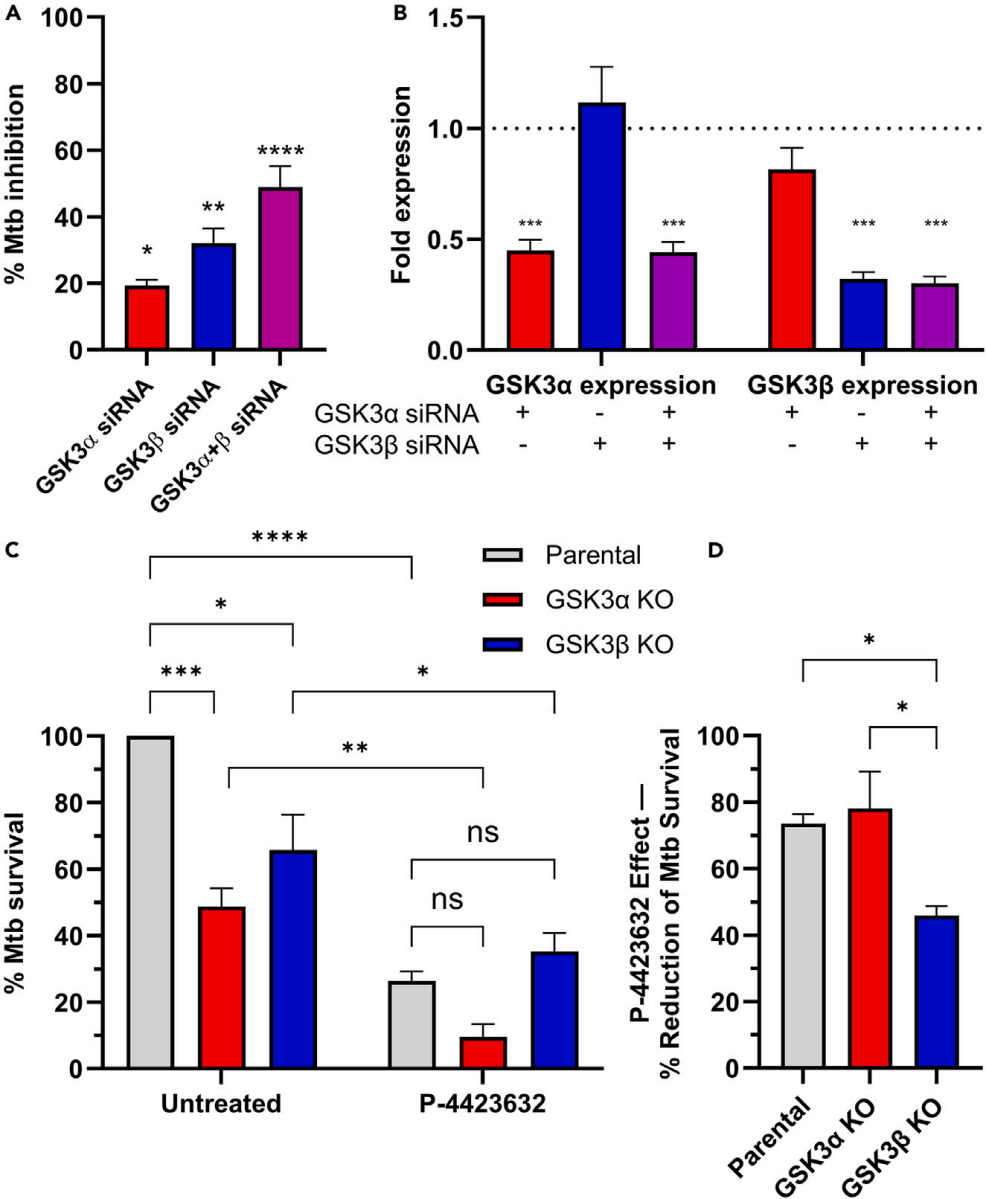
Genetic validation of GSK3’s role in restricting Mtb intracellular growth (A) Inhibition of intracellular Mtb by downregulating GSK3 isoforms using siRNA. GSK3α, GSK3β or both were knocked down in THP-1 cells, followed by infection with Mtb. Inhibition % represents the % of Mtb fluorescence area and relative luminescence compared to BDQ control (100% inhibition) and normalized to cells transfected with scrambled siRNA (0% inhibition). Data represent mean ± SEM of 3 biological replicates using high-content analysis (CX5) and luciferase assay; statistics were performed using one-way ANOVA followed by Bonferroni’s post hoc test compared to the scrambled siRNA control (not shown); ∗*p* < 0.05, ∗∗*p* < 0.01, ∗∗∗*p* < 0.001. (B) Confirmation using qPCR of siRNA knockdown of GSK3α and GSK3β RNA levels in THP-1cells following transfection with GSK3α siRNA (red), GSK3β siRNA (blue), and both GSK3α and GSK3β siRNA (purple); siRNA transfection is indicated. Fold expression represent the GSK3 variant expression levels in cells transfected with the indicated GSK3 siRNA compared to transfection with scrambled siRNA control, defined at 1 (not shown). Data represent the mean +SD of a representative experiment. *N = 4*. Statistics were performed using two-way ANOVA followed by Bonferroni’s post hoc test compared to the scrambled siRNA control; ∗∗∗*p* < 0.001. (C) Mtb infection of CRISPR-inactivated GSK3α (red) or GSK3β (blue) in THP-1 cells showing the effect of gene disruption with and without 72h P-4423632 treatment. Parental, GSK3α and GSK3β knockout THP-1 cells were infected with RFP-expressing Mtb and treated with 10 μM P-4423632 (right group) or DMSO control (Untreated, left group). Data represent high-content analysis of intracellular fluorescence area dual normalized to untreated (DMSO vehicle control) parental THP-1 cells (defined as 100% Mtb survival) and BDQ-treated parental cells (0% survival). Knocking out GSK3α and GSK3β in THP-1 cells were able to inhibit intracellular survival of Mtb with the greatest effect seen by blocking both GSK3α (by knockout) and GSK3β (by chemical inhibition). Data represent mean ± SEM of 3 biological replicates. Statistics were performed using two-way ANOVA followed by Bonferroni’s post hoc test. ∗*p* < 0.05; ∗∗*p* < 0.01; ∗∗∗*p* < 0.001; ∗∗∗∗*p* < 0.0001. (D) Contribution of P-4423632 inhibition of Mtb in parental, GSK3α and GSK3β backgrounds. Treating GSK3α KO cells with 10 μM P-4423632 further inhibited intracellular survival of Mtb by over 75% of its untreated counterpart, similar to the reduction observed in parental cells; whereas addition of P-4423632 to GSK3β KO cells had less of an effect (∼50% reduction). Reduction % was calculated as 100%-treated/untreated data from (C) for each cell type. Statistics were performed using one-way ANOVA. ∗*p* < 0.05; ∗∗*p* < 0.01.

As a large proportion of GSK3 inhibitors restricted intracellular growth of Mtb, we hypothesized that silencing or knocking down GSK3 expression would have a similar effect as chemical intervention, leading to a reduction in the growth of the tubercle bacilli within macrophages. Indeed, siRNA downregulation of GSK3α and GSK3β showed 30 and 40% inhibition of Mtb growth in macrophages, respectively (Figure 2A). When macrophages were transfected with siRNA against both GSK3α and GSK3β, more than 60% inhibition of Mtb inside macrophages was observed. Silencing GSK3α did not impact GSK3β transcription levels and vice versa (Figure 2B).

CRISPR inactivation of either GSK3α or GSK3β independently in THP-1 cells (Figure 2C, left group) confirmed these findings, showing about 50% and 30% reduction of intracellular Mtb 72 h post infection, respectively. Treatment with P-4423632 showed an additional inhibitory effect on Mtb intracellular growth in both CRISPR KO cell lines indicating that P-4423632 had some off-target activity in the GSK3β KO, most likely targeting the highly homologous GSKα isoform. Alternatively, P-4423632 can inhibit the bacterial growth, if the compound is concentrated inside macrophages. This is less likely, as P-4423632 is toxic at high concentrations in THP-1 cells (Table S3). Nevertheless, the contribution of P-4423632 inhibition of Mtb growth was significantly less in the GSK3β KO background (Figure 2D), with the greatest activity seen when GSK3α was knocked out by CRISPR and GSK3β was inhibited by P-4423632 (Figure 2C, right group), thus maximally inactivating both GSK3 isoforms. These results indicate that the primary target of P-4423632 is GSK3β, as designed, while both GSK3α and β participate in controlling the fate of Mtb infection. Together, these siRNA and CRISPR knockout experiments validated our chemical genetics studies and confirmed the key role of host GSK3α and β in controlling intracellular growth of Mtb in THP-1 cells.

### P-4423632 inhibits the intracellular growth of Mtb in primary human macrophages

**Figure 3:**
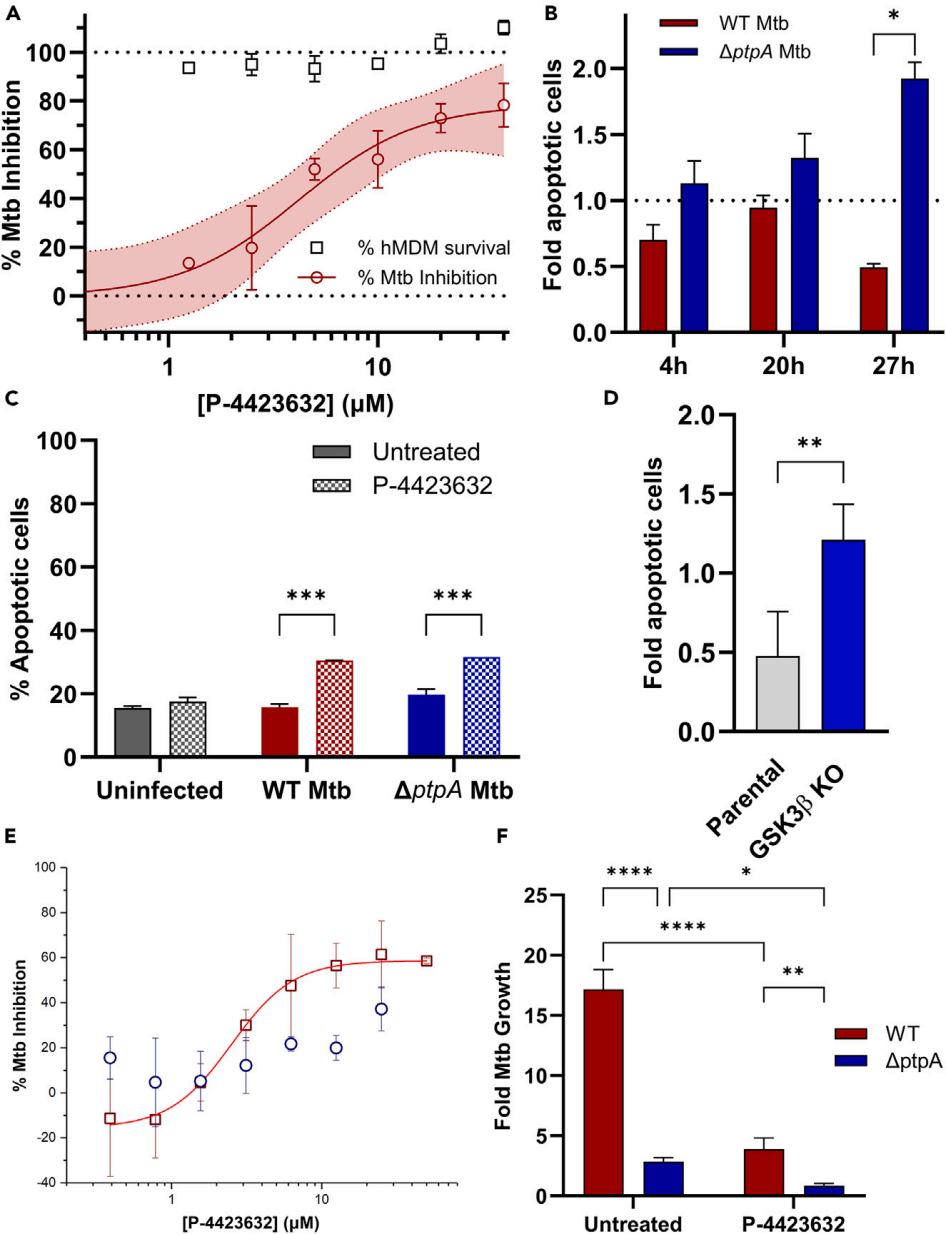
Inhibition of Mtb growth in primary macrophages and modulation of apoptosis (A) Dose-response curve of P-4423632 at 72 h post treatment of hMDMs infected with Mtb (dark red circles) and hMDM % survival (black squares). Data were acquired using the OPERA Phoenix High-Content microscope and analyzed using the Harmony software. Mtb inhibition at 72 h was calculated in relations to non-treated Mtb reflecting the negative % difference of intracellular Mtb fluorescence area compared to the DMSO vehicle control, normalized to the fluorescence area at 2h post infection. The % survival of hMDMs was calculated based on the nuclear count (DAPI stain) of cells compared to the DMSO control. Data represent 3 biological replicates ±SEM of mixed donors of the average of quadruplicate technical repeats. *N* = 3. Non-linear regression was used to fit the data of the log (inhibitor) vs. response (variable slope) curve using GraphPad Prism 10 analysis software. Shaded area represents the 95% confidence bands of the true curve. (B) Decreased THP-1 apoptosis during Mtb infection is associated with PtpA. THP-1 cells were infected with Mtb WT (dark red) or Mtb Δ*ptpA* (dark blue) at MOI of 6. Apoptotic activity was determined using the AUTOptosis method by monitoring chromatin condensation using DAPI staining. Data were normalized to uninfected cells and are a representative of three separate experiments ±SD; *N* = 6, ∗∗*p* values of 0.0022 performed by Mann Whitney non-parametrical test. (C) GSK3β inhibitor increases apoptotic activity in THP-1 cells. THP-1 cells infected with Mtb WT or Mtb Δ*ptpA* at an MOI of 3, with or without treatment with 20 μM P-4423632 and harvested at 48 h post-infection. Apoptotic activity was determined using Annexin V FITC assay. Statistical differences between treated and untreated groups were analyzed by two-way ANOVA followed by Bonferroni’s post hoc test; *N* = 2, ∗∗∗*p* < 0.001. (D) CRISPR-KO of GSK3β in THP-1 cells (blue) increased apoptosis in response to infection with Mtb compared to parental THP-1 cells (gray) in relation to non-infected cells. Apoptotic activity ±SD was measured using the AUTOptosis method; *N* = 6, ∗∗*p* values of 0.0043 performed by Mann Whitney non-parametrical test. (E and F) Inhibitory effect of GSK3β inhibitor, P-4423632, in PtpA knockout background. (E) Dose dependent activity of P-4423632 against intracellular Mtb (dark red squares) compared to the Δ*ptpA* mutant (dark blue circles), determined by high content analysis of fluorescent Mtb. Mtb % inhibition was calculated by dual normalization to the positive control and negative control ±SD. Non-linear regression (variable slope) was used to fit the data of the log (inhibitor) vs. response using GraphPad Prism software. (F) CFU counts of THP-1 cells infected with WT Mtb or Δ*ptpA* mutant with or without 96 h of 10 μM P-4423632 treatment. Data are a representative of two biological experiments performed in triplicate ±SD, *N* = 3. Statistical differences between treated and untreated, and WT and mutant were analyzed by two-way ANOVA followed by Bonferroni’s post hoc test; ∗∗∗*p* < 0.001, ∗∗*p* < 0.01, ∗*p* < 0.05.

To check whether P-4423632 is also active against intracellular Mtb in human primary cells we carried out two independent sets of experiments examining infected monocyte-derived macrophages (hMDM) obtained from normal human peripheral blood mononuclear cells (PBMCs) and monitored the infection over time using high content single cell imaging systems.^20^^,^^21^ As seen in Figures 3A and S5, analyzing Mtb growth in hMDMs combined from two donors, P-4423632 restricted Mtb intracellular replication in a dose dependent manner with no effect on host cell viability. Although some variability was observed in infection rates between three independent donors without treatment, a clear time and dose dependent antimicrobial activity of P-4423632 was observed (Figure S6). Interestingly, antimicrobial activity correlated with reduced variability of Mtb growth among donors and greater survival of macrophages over time (Figure S6).

### GSK3β inhibitors control intracellular Mtb via activation of host cell apoptosis

Apoptosis has been suggested to be a key mechanism by which host macrophages control Mtb infection.^22^^,^^23^^,^^24^^,^^25^ Indeed, we have shown that Mtb infection downregulates macrophage apoptotic activity in a PtpA-dependent manner (Figure 3B and ref.^11^). Treatment with P-4423632 resulted in upregulation of apoptosis (Figures 3C and S7) and CRISPR-KO of GSK3β in THP-1 cells increased apoptosis in response to infection with Mtb (Figure 3D). P-4423632 showed dose-dependent activity only against Mtb H37Rv but not against a Δ*ptpA* mutant (Figure 3E) and viable colony forming units (CFU) showed decreased effect against the mutant (Figure 3F). Comparative MIC analysis (Table S1) of selected GSK3 inhibitors against Mtb H37Rv and the Δ*ptpA* mutant confirmed these results, showing no detected inhibitory activity at the concentrations tested against the mutant while active at low micromolar concentrations against the parental strain. Together, these data reveal that both chemical inhibition of GSK3β, a target of Mtb PtpA, and its genetic deletion, result in the enhancement of host cell apoptotic response, associated with decreased intracellular survival of Mtb in THP-1 cells.

### P-4423632 interferes with host signaling pathways

**Figure 4:**
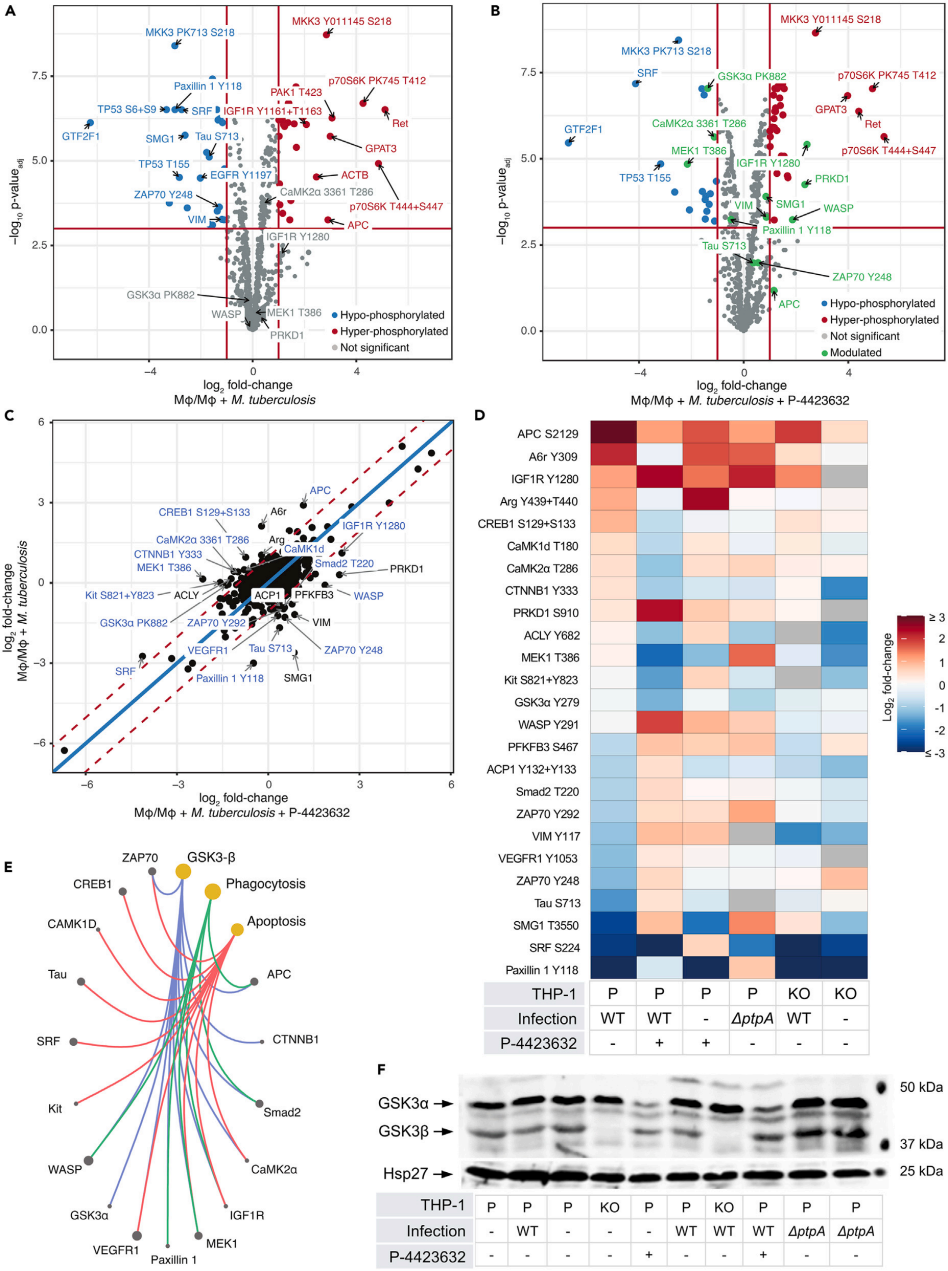
Modulation of macrophage phosphorylation upon infection and treatment with P-4423632 using antibody microarray analysis (A and B) Volcano plots showing log_2_ fold change in normalized signal intensity and log_10_*p* values for modulated protein phosphorylation sites after (A) infection with Mtb and (B) infection with Mtb and treatment with P-4423632. Red and blue labels show significantly up-regulated and down-regulated phosphosites, respectively. Green labels show phosphosites whose phosphorylation levels in relation to infection were noticeably modulated by P-4423632. *p* values were adjusted using the Benjamini-Hochberg method.^29^ (C) Scatterplot showing the ratio of log_2_ fold change in phosphorylation after infection or infection plus treatment with P-4423632. *p* values were calculated using an empirical Bayesian variance estimate derived from Cyber-T method.^30^ The solid blue line corresponds to a one-to-one ratio of log_2_ fold change while the red dotted lines represent the 97.5% quantiles of the data. The 25 proteins with the largest difference in fold change (distance to the blue line) are labeled in black. (D) Heatmap showing log_2_ fold change in relation to non-infected macrophages control. The 25 most modulated phospho-sites between infection and drug treatment were monitored in the labeled samples: P = parental THP-1 cells, KO = GSK3β CRISPR knockout in THP-1 cells, WT = infected with WT Mtb, Δ*ptpA* = infected with Δ*ptpA* mutant of Mtb. Row labels include the protein name and the affected phosphorylation site. (E) Network diagram 16 highly modulated proteins from (C) belonging to the GSK3β (blue lines), phagocytosis (green) lines, and apoptosis (red lines), related pathways. Pathways are defined by KEGG with GSK3β related disease specific pathways filtered out. Node and point size in the diagram reflect either the number of pathway groups a protein is present in or the number of proteins present in the pathway group. Visualization and statistical tests for antibody microarray analyses (A–E) were performed using CAT PETR.^31^ (F) Western blot analysis of Y279 - Y216 phosphorylation status of GSK3α and GSK3β cells infected with Mtb. P = parental THP-1 cells, KO = GSK3β CRISPR knockout in THP-1 cells, WT = infected with H37Rv WT Mtb, Δ*ptpA* = infected with Δ*ptpA* mutant of Mtb. Hsp27 included as loading control.

To identify potential substrates and or signaling pathways that are modulated by GSK3β, we monitored the kinome and phosphoproteome of THP-1 macrophages using a microarray of specific antibodies against the phosphosites of human signaling proteins (Kinexus Bioinformatics^26^). We focused on the effect of P-4423632 and GSK3β CRISPR KO on host macrophage signaling during infection with WT Mtb and Mtb Δ*ptpA* mutant (Data S1, S2, and S3). As observed previously,^11^ antibody microarray analysis of macrophage response to infection shows modulation of various signaling proteins and their corresponding phosphosites (Figure 4A). Mtb infected macrophages treated with P-4423632 showed a similar pattern, yet the phosphorylation levels of some key signaling proteins changed upon treatment (Figure 4B). Fold change analysis of untreated infected cells vs. infected cells treated with P-4423632 identified key signaling protein phosphosites that were affected by the drug (Figures 4C and 4D). Among these, several that were upregulated during infection, including the WNT signaling regulator, adenomatous polyposis coli,^27^ APC S2129; the actin binding protein twinfilin,^28^ A6R Y309; the Abelson murine leukemia viral oncogene, Arg Y439 + T440; and the cAMP-responsive element binding protein 1, CREB1 S129 + S133, were downregulated by treatment in the GSK3β CRISPR KO and in THP-1 cells infected with the Δ*ptpA* mutant (Figures 4C and 4D).

Several kinases did not change their phosphorylation status upon infection, but showed reduced phosphorylation during treatment. These include: the target of P-4423632, GSK3α Y279 (GSK3β homolog Y216); the proto-oncogene receptor tyrosine kinase, Kit S821 + Y823; Mitogen activated kinase-kinase MEK1 T386; ATP citrate lyase, ACLY Y682; catenin beta 1, CTNNB1 Y333; and the calcium calmodulin-dependent protein kinases, CaMK1d T180 and CaMK2a T286. Wiskott-Aldrich syndrome protein, WASP Y291^32^ and human serine threonine kinase PRKD1 S910 did not change upon infection, but showed increased phosphorylation with the addition of the drug.

The last set includes those protein phospho-sites whose phosphorylation level was reduced by infection but upregulated following treatment with P-4423632 (but not upregulated by the drug in the absence of infection) (Figure 4D). These include: the focal adhesion-associated protein, Paxillin Y118^33^; the phosphatidylinositol kinase (PIK)-related protein, SMG1 T3350^34^; tubulin associated unit protein, Tau S713; Zeta-chain-associated protein kinase 70, ZAP70 Y248^35^; vascular endothelial growth factor receptor, VEGFR1 Y1053; Smad2 T220; and acid phosphatase 1, ACP1 Y132 + Y133.

Network analysis of the identified phosphor-sites that were affected by P-4423632 (Figures 4C, 4E, and S8) identified two proteins (IGF1R and MEK1) that link GSK3β to both processes of phagocytosis and apoptosis. Four out of six identified proteins link GSK3β to the process of phagocytosis and five out of ten link apoptosis to phagocytosis. This demonstrated that the global kinome response to mycobacterial infection was affected by P-4423632 in part through GSK3β control of phagocytosed Mtb mediated by apoptosis.

GSK3α and β enzymes played a role in the control of cell fate via phosphorylation and dephosphorylation of Y279 and Y216, respectively. As seen in Figure 4F, infection of THP-1 cells with Mtb did not change the phosphorylation level of either residue. However, reduced phosphorylation of both Y279 and Y216 was observed compared to cells infected with an Mtb Δ*ptpA* mutant or treated with P-4423632, confirming results obtained by the antibody microarray analysis. Infection or treatment with P-4423632 did not affect the phosphorylation level of other reactive GSK3α and β phosphosites.

## Discussion

The novel anti-infective drug discovery approach termed HDT, was proposed recently to overcome drug resistance.^4^ Since HDTs do not target microbial pathogens directly but rather assist the host in fighting infection, they would have reduced chances of generating resistance. As such, HDT approaches prevent intracellular bacteria from thriving inside the human host. HDTs can synergize with antimicrobial chemotherapy and have been proposed for improving treatment outcomes and reducing the duration of therapy.^4^^,^^6^^,^^36^^,^^37^ The notion of HDT is supported by strong evidence that host signaling and immune responses play a critical role during TB pathophysiology, and that 90% of infected individuals have the innate capacity to overcome disease without treatment.^38^ Despite 90% recovering on their own, TB in the remaining 10% drives the highest death rate due to a single infectious agent; thus, better treatments are urgently needed.

Studies from almost 30 years ago showed that infection with Mtb prevented spontaneously occurring apoptosis in infected monocytes^39^ suggesting the hypothesis that macrophage apoptosis contributes to innate host defense in TB.^23^ Mtb infection was associated with lower apoptosis levels in macrophages compared to ones infected with *M. bovis*^40^ and Mtb survival in host macrophages involves induction of a signaling pathway promoting resistance to apoptosis.^24^ Furthermore, dysregulation of apoptotic genes upon infection suggest that apoptosis is a major functional pathway that could be targeted for host-directed therapeutics.^41^ Indeed, we and others showed that Mtb promotes its intracellular survival by downregulating apoptosis.^11^^,^^41^^,^^42^^,^^43^^,^^44^^,^^45^ Although the impact of cellular apoptosis in controlling Mtb during infection is uncertain.^46^ Stutz et al.^47^ provide compelling evidence that apoptosis controls Mtb infection *in vivo*. More recently MCL-1 and BCL-2 inhibitors were shown to induce apoptosis of Mtb-infected macrophages suggesting that targeting the intrinsic apoptosis pathway is a valid approach for TB host-directed therapy.^48^

GSK3 is a key signaling protein present in eukaryotes as two main isoforms, GSK3α and GSK3β; both isoforms control multiple cell metabolic processes.^16^^,^^49^ GSK3 has been shown to play a role in controlling viral replication including hepatitis C,^50^ human immunodeficiency virus,^51^ and herpes simplex virus.^51^ Recently, GSK3β gained renewed interest as a target for HDT, as it was shown to control SARS-CoV-2 infection via phosphorylation of the viral nucleocapsid (N) protein.^52^^,^^53^ In addition, phenotypic screening against *Plasmodia* parasites has uncovered GSK3 as a vulnerable kinase target in parasitic infections^54^ and GSK3β, specifically, has been implicated in the regulation of cytokine production and immune responses to bacterial and parasitic infections.^55^

Interestingly, evidence for the role of GSK3 in controlling Mtb infections is often contradicting. On the one hand, this study and our prior publication^11^ showed that Mtb promotes its intracellular survival by downregulating apoptosis through GSK3α. On the other hand, Zhou et al.,^56^ showed that GSK3α/β inhibition with SB216763 and gene silencing increased Mtb infection. Etna et al.^57^ showed that silencing or pharmacological inhibition of GSK3β resulted in disrupting the rapamycin-driven modulation of the pro- and anti-inflammatory cytokine balance, which indicates that in Mtb-infected dendritic cells, GSK-3β acts as a molecular switch for the regulation of the cytokine milieu. Indeed, it was shown earlier that inhibition of GSK3 has contradicting effects that either promote cell death or inhibit it^16^ depending on whether the intrinsic or extrinsic apoptotic pathway is activated.^58^ Infection studies of murine peritoneal macrophages showed that Sirtuin, an apoptosis resistance associated gene, contributes to Mtb replication within these macrophages.^42^ Addition of the GSK3β inhibitor, TSW119, restores Mtb replication within Sirt^+/−^ cells,^42^ indicating a role for GSK3β in controlling apoptosis within infected cells and provides further support for our findings. Overall, the interactions between the host macrophage and Mtb reflect a delicate balance between bacterial growth and host cell functionality throughout infection. Host-pathogen interactions are dynamic and prevention of apoptosis can be used to curtail infection once this balance is disrupted by either microbial out-growth or damage to host macrophages. Apoptotic macrophages containing Mtb can be re-captured by circulating macrophages and in line with the observation that apoptosis and not necrosis is coupled with killing of intracellular bacteria,^59^ studies suggest that efferocytosis contributes to disease control.^60^^,^^61^

In this study, we found that a large set of GSK3 inhibitors are active against intracellular Mtb and specific compounds targeting GSK3β significantly decreased the number of replicating Mtb intracellularly in a THP-1 macrophage model. Inhibition of GSK3β resulted in host cell’s dependent antimicrobial activity against intracellular bacteria. The low μM activity against intracellular Mtb is an unusual characteristic of proposed adjuvant HDT. The observed limited activity of P-4423632 against the Δ*ptpA* mutant strain indicates, as previously suggested^11^ that GSK3β control of infection is mediated through PtpA. However, this observation poses limitations in conducting animal trials in mice as PtpA is not required for growth in mice.^62^ Genetic validation using gene silencing and CRISPR knockout of both GSK3α and GSK3β in THP-1 cells, demonstrate the role of both genes in controlling early infection of Mtb in these monocytic like macrophages. Chemical inhibition of Mtb growth in hMDMs extends this observation to primary human macrophages and suggests that inhibition of GSK3β might be linked to the synchronization or control of infected macrophages death (Figure S6).

The availability of specific inhibitors, gene knockout strains and an array of specific phosphorylation signaling antibodies enabled us to carry out a GSK3-dependent network analysis in infected macrophages. This phospho-proteome analysis identified and strengthened the link between phagocytosed Mtb, apoptosis and GSK3β, and confirmed the apoptosis phenotype associated with Mtb and specifically its secreted phosphatase PtpA. Furthermore, in addition to GSK3, two of the effected signaling proteins identified in our kinome analysis, VEGFR and IGF1R were identified as viable targets for the control of Mtb infection (Figure S1). Two out of eight PKIS inhibitors targeting IGF1R showed between 27% and 42% inhibition of intracellular Mtb (Figure S1) indicating that IGF1R is downstream of GSK3β.

To conclude, our results imply that GSK3β can be used as a *bona fide* target HDT against TB and further adds to our understanding of the signaling mechanisms that govern the intracellular growth phenotype of Mtb. Although limited to human macrophages, in our study, the high efficacy of GSK3β inhibitors in an *ex vivo* infection model, together with our genetic validation studies, merit serious consideration of further development of HDTs against intracellular pathogens such as Mtb, as alternative or supplemental therapies to antibiotics.

### Limitations of the study

Although the GSK3β inhibitor that controls Mtb intracellular growth is highly effective at the cellular level, organoid and animal studies still need to be performed in order to progress into clinical development studies. The inhibitor possesses some off-target activity, most likely targeting GSK3α, but the contributions of this activity were not fully elucidated, nor were the exact effects of the inhibitor on GSK3 α/β global phosphorylation status. Although our study has identified GSK3 as a key host-directed target for the potential treatment of tuberculosis, and while we identified apoptosis of infected macrophages as an affected pathway, further studies are needed to provide a more detailed mechanism of action.

## STAR★Methods

### Key resources table

**Table:** 

REAGENT or RESOURCE	SOURCE	IDENTIFIER
**Antibodies**		

Rabbit Phospho-GSK3A/B PY279/PY216	Invitrogen	Cat# 44-604G
Rabbit GSK3B Polyclonal antibody	ProteinTech	Cat# 22104-1-AP
Mouse anti β-actin BA3R	Invitrogen	MA5-15739
Goat anti-rabbit IgG-Peroxidase antibody	Sigma-Aldrich	A6154
Goat anti-mouse IgG-HRPO antibody	Cedarlane	Cat# CLCC30007

**Bacterial and virus strains**		

*M. tuberculosis* H37Rv	Av-Gay Lab stock, University of British Columbia	N/A
*M. tuberculosis* H37Rv pJAK2.A expressing luciferase	Plasmid (64) transformed (71)	N/A
*M. tuberculosis* H37Rv pTEC27 expressing RFP	(68)	RRID:Addgene_30182 (63)
*M. tuberculosis* H37Rv pTEC19 expressing E2 Crimson	(65)	RRID:Addgene_30178 (63)
*M. tuberculosis* H37Rv pFPV2 expressing GFP	Plasmid: (72), transformed (66)	ATCC 27294
*C. jejuni*	(73)	81-176
*Acinetobacter baumannii*	ATCC	ATCC# BAA-747
*Bacillus subtilis*	ATCC	ATCC# 6633
*Escherichia coli*	ATCC	ATCC# 25922
*Enterococcus faecalis*	ATCC	ATCC# 29212
*Moraxella catarrhalis*	ATCC	ATCC# 25240
*Pseudomonas aeruginosa*	ATCC	ATCC# 14210
*Staphylococcus aureus*	ATCC	ATCC# 25923
*MRSA*	ATCC	ATCC# 700698
*Staphylococcus epidermidis*	ATCC	ATCC# 35984
*Salmonella typhimurium*	ATCC	ATCC# 13311
*Mycobacterium marinum*	Vancouver Collection	JVC 1704
*Mycobacterium intracellulare*	ATCC	ATCC# 35761
*Mycobacterium bovis* BCG	Pasteur	Pasteur 1173P2
*Mycobacterium smegmatis*	Albert Einstein College of Medicine	mc^2^155
*Mycobacterium abscessus*	ATCC	ATCC# 19977T (R)
*Mycobacterium tuberculosis H37Rv auxotroph,* mc^2^ 6206	Albert Einstein College of Medicine	mc^2^6206
*M. avium avium*	Av-Gay Lab stock, University of British Columbia	N/A

**Biological samples**		

Leukocyte cones	UK National Health Service	NC24
Blood (leukocyte concentrate)	Swedish healthy blood donors	Linköping University Hospital

**Chemicals, peptides, and recombinant proteins**		

Middlebrook 7H9	BD	Cat# CA90003-876
Bovine Serum Albumin	Sigma Aldrich	Cat# A7906
Dextrose anhydrous	Fisher BioReagent	Cat# BP350-1
Catalase	Sigma Aldrich	Cat# c9322
Oleic acid	Sigma Aldrich	Cat# O1008
RPMI1640	Sigma Aldrich	Cat# R5886
L-glutamine	Sigma Aldrich	Cat# G7513
NEAA	Gibco	Cat# 11140-050
1% penicillin and 1% streptomycin	Gibco	Cat# 15140-122
DMEM	Sigma-Aldrich	Cat# D5796
Human Serum	Sigma-Aldrich	Cat# H4522
Fetal Bovine Serum, qualified, heat inactivated	ThermoFisher	Cat# 12484028
PBS	Gibco	Cat# 14190144
Phorbol12-myristate13-acetate	Sigma Aldrich	Cat# P1585
Paraformaldehyde	ThermoFisher	Cat# 28908
Hoechst 33342, Trihydrochloride, Trihydrate	ThermoFisher	Cat# H1399
Thiazolyl Blue Tetrazolium Bromide (MTT)	Sigma Aldrich	Cat# M2128
Resazurin Sodium Salt	MP Bio	Cat# 194598
Bright-Glo luciferase reagent	Promega	Cat# E2610
HiPerFect transfection reagent (Qiagen)	Qiagen	Cat# 301705
FastStart Universal SYBR Green Master (Rox)	Roche	Cat# 4913850001
DMSO	Fisher Scientific	Cat# BP231
Bedaquiline	ApexBio	Cat# B3492-10
Rifampicin	BioWorld	Cat# 41810012-2
Kanamycin	Fisher Scientific	Cat# BP906
Hygromycin B	Roche	Cat# 10843555001
Published Kinase Inhibitor Set (PKIS)	University of North Carolina at Chapel Hill (67)	N/A
GSK3β-inhibitor library	Takeda Pharmaceutical Company (17, 18)	N/A
PBS [-]CaCl_2_,[-]MgCl_2_,pH 7.2	Gibco	Cat# 14190-094
PBS EDTA	Sigma	Cat# E5134
autoMACS buffer	Miltenyi Biotec	Cat# 130-091-222
BSA	Cell Signaling Technology	Cat# 9998S
Ficoll-Paque	GE Healthcare Bio-Sciences AB	Cat# 17-5442-02
RBC lysis buffer	Sigma	Cat# R7757
RPMI (w GlutaMAX and 25mM HEPES) +	Gibco	Cat# 72400-021
FCS	Cell Services (Crick)	N/A
CD14 microbeads, human	Miltenyi Biotec	Cat# 130-097-052
LS columns	Miltenyi Biotec	Cat# 130-042-401
Tripan blue solution	Sigma	Cat# T8154-100ML
Lymphoprep	StemCell Technologies	Cat# 07801
dPBS	Gibco	Cat#14190-144
FBS (heat inactivated)	Gibco	Cat#A5256701
Normal human serum	pooled from 5 Swedish healthy blood donors	N/A
L-glutamin	Gibco	Cat#25030-024
Difco Middlebrook 7H9 broth	BD Bioscience	Cat#271310
Tween-80	Sigma-Aldrich	Cat#P8074
glycerol	VWR	Cat#24385.295
kanamycin	Sigma-Aldrich	Cat#B5264
BBL™ Middlebrook ADC Enrichment	BD Bioscience	Cat#211887
draq7	BD Bioscience	Cat#564904
DMSO	Sigma-Aldrich	Cat#D5879
Rifampicin	Sigma-Aldrich	Cat#R3501
Isoniazid	Sigma-Aldrich	Cat#I3377
Bovine Serum Albumin Fraction V	Roche	Cat# 10735078001
Sodium Chloride	Fisher Scientific	Cat# S271-3
Tris Base	Fisher Scientific	Cat# BP152-1
Tween 20	Fisher Scientific	Cat# BP337-100
Hydrochloric Acid 37%	Sigma Aldrich	Cat# 435570
SuperSignal West Pico PLUS Chemiluminescent Substrate	ThermoFisher	Cat# 34577

**Critical commercial assays**		

Illustra RNAspin minikit	GE Healthcare	Cat# 25-0500-71
OneScript® Plus cDNA Synthesis Kit	abm	Cat#G236
QIAamp 96 DNA Kit	Qiagen	Cat# 51331
Geneclean III kit	MP Biomedicals	Cat# 111001600
FITC Annexin V Apoptosis Detection Kit I	BD BioSciene	Cat# 556547

**Experimental models: Cell lines**		

THP-1 (male)	ATCC	ATCC® TIB-202; RRID:CVCL_0006
THP-1 GSK3β KO (male)	This paper	N/A
THP-1 GSK3α KO (male)	This paper	N/A
Caco-2 (male)	ATCC	ATCC® HTB-37; RRID:CVCL_0025

**Oligonucleotides**		

siRNA targeting sequence: Scrambled (Negative Control DsiRNA)	IDT	Cat# 51-01-14-04
siRNA targeting sequence: GSK3β	IDT	hs.Ri.GSK3B.13.3
siRNA targeting sequence: GSK3α	IDT	hs.Ri.GSK3A.13.2
GSK3α CRISPR Guide Sequence: GCCUAGAGUGGCUACGACUG	Synthego	N/A
GSK3β CRISPR Guide Sequence: CGUUAUUUCUUCUACUCCAG	Synthego	N/A
GSK3α CRISPR Sequencing For primer: CCTTCTCTTCTGGCCCATGG	Synthego	N/A
GSK3α CRISPR Sequencing Rev primer: CAACGAGCTTCCTGCAGAGA	Synthego	N/A
GSK3β CRISPR Sequencing For primer: TTCAGAAGCATCTTGTTCAATATG	Synthego	N/A
GSK3β CRISPR Sequencing Rev primer: CAAGTGTTTCGGAATTTTTCC	Synthego	N/A
GSK3β CRISPR Sequencing Rev primer: CAAGTGTTTCGGAATTTTTCC	Synthego	N/A
Primers for qPCR, see Table S4	This paper	N/A

**Software and algorithms**		

Graph Pad Prism v10	Dotmatics https://www.graphpad.com/scientific-software/prism/	RRID:SCR_002798
HCS Data Explorer	Manuscript in preparation	N/A
CAT-PETR	(62)	N/A
Microsoft Excel 2019 for Windows v1808	Microsoft	RRID:SCR_016137www.microsoft.com/en-ca/microsoft-365/excel

### Resource availability

#### Lead contact

Further information and requests for resources and reagents should be directed to and will be fulfilled by the lead contact, Yossef Av-Gay (yossi@mail.ubc.ca).

#### Materials availability

There are no restrictions to the availability of materials.

#### Data and code availability

• All data reported in this paper will be shared by the lead contact on request.
• This paper does not report original code.
• Any additional information required to reanalyze the data reported in this paper is available from the lead contact upon request.

### Experimental model and study participant details

#### Bacterial cultures

WT Mtb H37Rv, H37Rv harboring pTEC27 (RFP),^63^ pJAK2.A (luciferase^64^), pTEC19 (E2 Crimson),^65^ or pFPV2 (GFP), and H37Rv Δ*ptpA* cultures were maintained at 37°C in Middlebrook 7H9 broth supplemented with 10% OADC or ADC, 0.05% Tween 80 and appropriate antibiotic(s).

#### Cell lines

The human monocytic THP-1 (TIB-202; ATCC) cell line was maintained at humidified 37°C and 5% CO_2_ in RPMI 1640 medium supplemented with 10% FBS, 2% L-glutamine, and 1% penicillin and 1% streptomycin for culture expansion. For cell differentiation, THP-1 cells were seeded in a 96 well plate (1.0 x 10^5^ cells/well) or 100 mm petri dish (7.5 x 10^7^ cell/dish) and incubated overnight at 37°C and 5% CO_2_ with 20 ng/ml of phorbol myristate acetate. For differentiation and infection, cells were maintained in antibiotic-free media.

#### Primary cells

Human MDMs were prepared from two donor blood cones supplied by the NHS Blood and Transplant service as described previously^21^ and used in Figures 3A and S5. Briefly, monocytes were extracted in parallel from each donor and cells were combined for a total of 1.2 x 10^8^ cells. Monocytes were differentiated by addition of 10 ng/mL hGM-CSF (Miltenyi, 130-093-867) in RPMI 1640 with GlutaMAX and HEPES (Gibco, 72400-02), 10% foetal bovine serum (Sigma, F7524) and plated in 9 cm petri dishes at 1.2 x 10^6^ cells/mL in 10 mL per dish. Cells were incubated at 37°C with 5% CO_2_ for 6 days with a fresh media change including hGM-CSF after 3 days.

Peripheral blood mononuclear cells (PBMCs) were isolated from the blood of three healthy donors using SepMate (StemCell Technologies) following the manufacturer protocols as described previously^20^ and used in Figure S6. Human MDMs were isolated from PBMCs as described previously.^66^ Briefly, PBMCs were allowed to adhere for 2 h before non-adherent cells were washed away. The adherent monocytes were allowed to differentiate for 7 days in complete DMEM containing 25 mM HEPES, 100 U/ml penicillin, 100 μg/ml streptomycin, 2 mM L-Glutamine (Gibco) and 10% active human serum with media changes every 2-3 days.

### Method details

#### THP-1 cell infections with Mtb

Differentiated THP-1 cells were washed three times with RPMI 1640 medium. Mtb cultures were washed three times with Middlebrook 7H9 broth supplemented with 0.05% Tween 80 and opsonized with 10% human serum to prepare for infection. THP-1 cells were infected with Mtb at the indicated multiplicity of infection (MOI) in RPMI 1640 medium and incubated for 3 h at 37°C and 5% CO_2_. After the incubation period, THP-1 cells were washed three times with RPMI 1640 medium to remove non-internalized bacteria.

#### High-content intracellular screening

The GSK3 inhibitor library was kindly provided by Takeda Pharmaceutical Company Limited (Osaka, Japan).^17^^,^^18^ The Published Kinase Inhibitor Set (PKIS) was obtained from the University of North Carolina (Chapel Hill, NC, United States).^67^

A fluorescence-based high-content screening assay was used to screen compounds against Mtb as described previously.^68^ Differentiated THP-1 cells in 96-well plates were infected with Mtb pTEC27at MOI 2:1. After the 3-hour time point infection, compounds were added at a single concentration of 10 μM in RPMI 1640 medium and the plates were incubated for 72 h at 37°C and 5% CO_2_. After the 72-hour incubation, THP-1 cells were washed three times with RPMI 1640 medium and then the fluorescent dye DAPI (4′,6-diamidino-2-phenylindole) or Hoechst was added to stain the macrophage nuclei. After staining, the cells were fixed with 4% formaldehyde and the plate was read with the CellInsight™ CX5 high content screening platform (Thermo Fisher Scientific, Waltham, MA). Channel 1 identified DAPI fluorescence for focusing and counting viable THP-1 cells. Channel 2 measured the total and average area and intensity of the fluorescence signal intensity (RFU) of the bacteria. The fluorescence measurements of the bacteria were normalized to the negative control (1% DMSO), defined as 100% growth and the positive control (10 μM rifampicin or 4 μM BDQ, Figure S10), defined as 0% growth, in relation to a known antibiotic. The DAPI fluorescence signal was used to count the number of cells per well. These values were normalized to the DMSO negative control to determine the percentage of viable THP-1 cells.

#### High-throughput intracellular screening

The HTS was performed as described previously.^14^^,^^19^ THP-1 cells were seeded into 96-well plates and infected with a luciferase-expressing strain of Mtb at an MOI of 5:1. THP-1 cells were treated in triplicate with each GSK3 inhibitor for 72 h at 37°C and 5% CO_2_. After the 72-hour incubation period, THP-1 cells were washed once with PBS, and 50 μl of luciferase assay reagent were added to each well. After 5 min, the luminescence signal from the bacteria in each well was measured with a luminometer (Synergy™ HT plate reader).

#### Cytotoxicity analysis (MTT assay)

The MTT assay was performed as described previously.^19^ THP-1 cells were differentiated overnight and then treated with each GSK3 inhibitor for 72 h at 37°C and 5% CO_2_. At the 69.5-hour time point, 25 μL of 3-(4,5-dimethylthiazol-2-yl)-2,5-diphenyltetrazolium bromide (MTT) solution were added to each well. At the 72-hour time point, 100 μL of MTT extraction buffer were added to each well and incubated overnight at 37°C and 5% CO_2_. The absorbance of each well was read at 570 nm and these values were used to calculate the percentage of viable cells.

#### In-broth activity analysis (resazurin assay)

The Resazurin assay was performed as described previously.^14^^,^^19^ An Mtb culture was grown to mid-log phase. The bacterial culture was washed three times with Middlebrook 7H9 broth supplemented with 0.05% Tween 80, 10% (ADS) and diluted to an OD_600_ of 0.01. The bacteria were transferred to 96-well plates in 100 μL aliquots and treated in triplicate with each GSK3 inhibitor at 2x the testing concentration. The plate was incubated for 5 d at 37°C and 5% CO_2_. After the 5-day incubation period, 30 μL of resazurin solution were added to each well and the plate was incubated for an additional 48 h.

#### Determination of IC_50_ of GSK3 inhibitors

Samples of three GSK3 inhibitors (P-4423632, P-0717632, P-3817632) were sent to SignalChem (Richmond, BC, Canada) where a compound selectivity assay was performed and the IC_50_ values of the inhibitors were determined.

#### Dose dependency activity analysis of GSK3 inhibitors

THP-1 cells (10^5^ cells/well) were differentiated and infected with luciferase-expressing Mtb and treated in triplicate with GSK3 inhibitors at 2-fold serial dilutions from 20 μM to 0.156 μM for 72 h at 37°C and 5% CO_2_. After the 72-hour incubation period, THP-1 cells were washed once with PBS, and 50 μl of luciferase assay reagent were added to each well. After a 5-minute incubation, the luminescence signal from the bacteria in each well was measured with a luminometer (Synergy™ HT plate reader).

#### Infection of primary macrophages

Differentiated hMDMs were then detached, counted, and reseeded at 5 × 10^4^ cells per well in an olefin-bottomed 96-well plate (Perkin Elmer, 6055302) 16–20 h prior to infection as described previously^21^ Mtb harbouring the pTEC19 E2-Crimson-expressing plasmid were grown to OD_600_ ∼0.8. Bacteria were washed twice with PBS buffer (pH 7.4) and resuspended in a small volume of PBS and used in experiments for Figures 3A and S5. Bacteria were declumped by addition of 6-8 sterile glass beads (2.5-3.5 mm) with 1 min shaking and 1 min vortex. Cell culture media was then added and remaining clumps were pelleted by slow-speed centrifugation at 1200 x g for 5 min. Supernatant was collected and OD_600_ read with conversion factor of OD_600_ 1 = 10^8^ bacteria. Macrophages were then infected with Mtb at MOI 1 for 2 hours. Cells were washed and cell culture media containing 2-fold serial dilutions of P-4423632 were added in quadruplicate with DMSO vehicle control. Cells were fixed with 4% PFA at 2h and 72h time points post infection, stained with DAPI and imaged on the Phoenix Opera high content microscope using the 40x water-immersion 1.1 NA objective followed by analysis using the Harmony software (Perkin Elmer, version 4.9) as described previously.^21^

Macrophages were harvested by trypsinization and seeded at 5000 cells/well in 30 μL of the above antibiotic-free (ABF) media in a 384-well plate and incubated overnight. Mtb H37Rv expressing GFP (pFPV2) was prepared as described previously^66^ and used to infect the hMDMs in equal volume at MOI 2 for 3 h. Compounds were diluted in ABF media containing 0.3 mM Draq7 (BD Biosciences) at five times final concentrations and added to infected hMDMs to a final volume of 75 μL per well. A combination of Rifampicin and Isoniazid at 1 μg/mL each was used as a positive control for Mtb growth inhibition and 0.1% DMSO was used as the negative, vehicle control. All incubation steps were performed in a humidified environment at 37°C with 5% CO_2_. Infected hMDMs were monitored (Figure S6) using the Incucyte S3 (IncuCyte Live-Cell Analysis System, Sartorius) for a period of 14 days post-infection with images taken every 8 h (10x, 2 images/well). Bacterial growth was measured based on the area of relative fluorescence (GFP) signal. Cell death was measured based on RFP spot count of the Draq7 dye.

#### Apoptosis assay using AUTOptosis

Apoptosis was assessed using the AUTOptosis method^69^ and analyzed with CellInsight CX5 HCS platform (Thermo Fisher Scientific).

#### Annexin V assay

THP-1 cells (5 x 10^5^ cells/well) were differentiated overnight and then infected with WT Mtb, Δ*ptpA* Mtb. The infected THP-1 cells were washed three times with RPMI 1640 medium and harvested by adding cold PBS to each well. The cells were stained with FITC Annexin V and Fixable Viability Stain 570 (BD Horizon) in Annexin V Binding Buffer according to the manufacturer’s instructions. The cells were fixed with 2% formaldehyde in Annexin V Binding Buffer, washed and resuspended in Annexin V Binding Buffer. The resuspended cells were analyzed by flow cytometry using a BD FACSCanto II instrument (BD Biosciences, San Jose, CA) as described previously.^70^

#### Caco-2 infection with *C. jejuni*

Isolated colonies from overnight culture grown on Muller -Hinton (MH) plates in microaerophilic conditions were inoculated into liquid MH media and grown overnight. Bacteria were pelleted by centrifugation and resuspended in DMEM media. The bacteria were then added at an MOI of 100-400 to Caco-2 cells in 24-well plates at 10^5^ cells per well. After 2 h of incubation, infected cells were washed with fresh DMEM and incubated an additional 2 h with 100-200 μg/ml gentamycin followed by the addition of test compounds and incubation for 24 h. After incubation, cells were washed with fresh DMEM and lysed by osmotic shock with sterile ddH_2_O_2_ and passed through a 27G needle with a syringe. The lysate was serially diluted and plated in MH agar using the pouring plate method. CFU were then counted after a 24 - 48 h incubation. To assess cell survival, a duplicate of the previous conditions was used to undergo MTT assay or cell count with the CX5 using DAPI staining.

#### THP-1 infection with *S*. *typhimurium*

THP-1 cells were grown in complete RPMI1640 medium (5% FBS, 2% glutamine, 1% non-essential amino acids). Cells were grown in T75 flask with 5% carbon dioxide (CO2) at 37°C. Cell density was kept between 0.25 and 1 × 10^6^ cells/mL. Cultures were used for up to three months. A day before infection, THP-1 macrophages were seeded at 1 × 10^5^ cells per well in a 96-well plate with PMA (40 ng/mL). *Salmonella typhimurium* transformed with a fluorescent reporter plasmid, was grown overnight on a Luria-Bertani (LB) agar plate. A broth culture was started several hours prior to the infection and harvested once it read 1 at OD 600_nm_ to determine an MOI = 10 (1 x 10^6^ CFU per well). Bacteria were pelleted by centrifugation and washed three times with RPMI media. The bacteria were then opsonized for 30 min at 37°C with 10% human serum. The opsonized bacteria were diluted in RPMI to have 40 μL infection volume/well. After 30 min of incubation, the infected cells were washed three times with fresh RPMI and incubated for an additional hour with 100 μg/mL of gentamicin to kill remaining extracellular bacteria. The infected cells were incubated with the tested compounds in presence of 20 μg/mL of gentamicin. After 24h of infection, cells were washed twice with PBS and the intracellular growth of the bacteria was assessed using the High Content Screening platform CX5 (ThermoScientific). Bacterial growth was normalized to a non-infected control, and to an infected, untreated control.

#### siRNA mediated gene silencing of *GSK3*

THP-1 cells were seeded at 50,000 cells/well in 96-well plates and differentiated as described above. Following differentiation, the THP-1 cells were transfected using 10 pmole siRNA (IDT) and 2 μL of HiPerFect transfection reagent per well, according to the manufacturer’s instructions. THP-1 cells transfected with scrambled siRNA were used as a negative control. After a 24-hour incubation of the transfected cells at 37°C and 5% CO_2_, the cells were washed and infected with tdTomato-expressing Mtb as described above at MOI 2:1. After the 72-hour incubation period, infected THP-1 cells were washed, stained with Hoechst and fixed with 4% formaldehyde as described above. The plate with fixed cells was read with the CellInsightTM CX5 high content screening platform as described above. qPCR and Westerns detecting GSK3 variants transcripts and protein levels respectively are provided in Figure S9.

#### CRISPR interference of *GSK3 variants* in THP-1

We performed CRISPR interference in THP-1 cells to evaluate the effect of GSK3 gene deletions on the intracellular growth of Mtb. CRISPR knockouts in THP-1 cells were designed and synthesized at Synthego Corporation (Menlo Park, CA, USA) using single guide RNA (sgRNA) that target GSK3β and GSK3α genes. sgRNA and Cas9 were transfected into THP-1 cells and generated two cell pools containing WT cells and various mutations in the individual GSK3β and GSK3α genes. We isolated separate GSK3β and GSK3α KO clones from each pool through the limiting dilution method and then clonally expanded the candidate cells. Clones were analysed by PCR followed by sequencing and Western analysis. The limiting dilution method was used to isolate single cell THP-1 GSK3β KO clones and GSK3α KO clones from each cell pool as follows. The concentration of the cell suspension (cells/ml) was calculated and was diluted in 10-fold dilutions to a final concentration of 0.5 cells/100μl of medium. The diluted culture was dispensed into two 96-well plates by adding 100μl of culture to each well. The 96- well plates were incubated at 37°C and 5% CO_2_ and cell cultures were expanded as needed. Genotyping CRISPR clones by PCR and sequencing was used to determine whether the isolated clone had the desired knockout. To genotype the clones, DNA from multiple expanded cultures was isolated using the GeneJET Genomic DNA Purification Kit according to the manufacturer’s instructions (Life Technologies), followed by PCR amplification of the genetically altered region of the genome, and sequencing by GENEWIZ using Sanger sequencing. Sequence data were analyzed using SnapGene. Westerns detecting GSK3 variants protein levels are provided in Figure S9.

#### Quantitative real-time PCR

THP-1 cells (3 to 5 x 105) were transfected as above with siRNA for 24 h, washed and incubated for an additional 72h to mimic infection time. RNA was then extracted, combined and cleaned using the Illustra RNAspin Mini RNA Isolation Kit (GE Healthcare) according to the supplied protocol. Reverse transcription reactions were carried out in 20-μL volumes containing 250 ng RNA, oligo dT primers, and the buffer and enzyme components of the OneScript® Plus cDNA Synthesis Kit (ABM) according to the supplied protocol. Real-time PCR analysis was carried out on the CFX96 Real-Time System (BIO-RAD). Real-time qPCR reactions were carried out in 20-μL volumes containing 2X FastStart SYBR green master mix (Roche), 5 μL of 5-fold diluted cDNA and 0.3 μM of each primer. Control reactions without reverse transcriptase were included with each run to confirm the absence of genomic DNA contamination. Relative-fold expression levels were calculated using the delta-delta Ct method normalized to the GAPDH gene. All primers were ordered from IDT.

#### Western analyses

THP-1 cells (1.2 x 10^6^) were transfected as above in two wells of a 12-well plate with siRNA for 24 h, washed and incubated for an additional 72 h to mimic infection time. Cells were washed and lysates were collected on ice as well as lysates from differentiated CRISPR KO and parental THP-1 cells (6 x 10^6^). Sample buffer (8x) was added to protein samples and heated to 95°C for 5min. Samples were run on 10% SDS-PAGE and then transferred onto PVDF membranes using standard semi-dry transfer method by manufacture (Biorad, 15V for 30min). PVDF membranes were blocked using 3% BSA dissolved in Tris buffered Saline (TBS) for 1 hr at room temperature using a platform shaker. Membranes were washed 3x 10 min with TBS with 0.5% Tween-20 (TBST), followed by incubation with primary antibody diluted in TBST (anti-GSK3α, 1:500 dilution; anti-GSK3β, 1:5000; anti-β-actin, 1:10000) overnight at 4°C on a platform shaker. Membranes were washed 3 x 10 min with TBST and then incubated with secondary antibody diluted in TBST (Goat anti-rabbit, 1:5000; goat anti-mouse, 1:5000) for 1 hr at room temperature on a platform shaker. Membranes were washed 3x 10 min with TBST and then incubated with ECL reagent with shaking for 5 min followed by imaging using the Azure 300 Imager (Azure Biosystems).

#### Antibody microarrays analyses

Lysates were prepared from THP-1 cells (0.5 x 10^6^ cells/well) that were differentiated overnight and then infected with WT Mtb and Δ*ptpA* Mtb at MOI 10:1 and treated with or without 10 μM P-4423632. Lysates were subjected to Kinexus Kinex™ KAM-2000 antibody microarray analyses as described.^26^ The KAM-2000 microarrays utilized 2059 commercial, pan-specific antibodies for 939 non-redundant human protein targets that included protein kinases, phosphatases, transcription factors, stress proteins and many other signaling proteins. The KAM-2000 microarray featured 1165 pan-specific and 894 phosphosite-specific commercial antibodies, produced principally by Kinexus Bioinformatics (Vancouver, BC, Canada) as well as from other suppliers following their in-house validation, with each antibody printed in quadruplicate on each Nexterion P slide (Schott AG, Jena, Germany). Briefly, cells were scrapped in Standard Homogenizing Buffer [1% Triton X-100, 5 mM EDTA, 2 mM EGTA, 20 mM MOPS, 25 mM NaF, 25 mM Na_4_P_2_O_7_, 1.0 mM Na_3_VO_4_, 60 mM beta-glycerophosphate, 50 nM phenylarsine oxide, 1 mM Pefabloc, 3 mM benzamidine, 10 μM leupeptin, 0.5 μM aprotinin, 1 mM dithiothreitol, 100 mM NaCl] and 400 mM Tris (2-carboxyethyl) phosphaine hydrochloride (TCEP) at pH 9.0 (to reduce disulfide linkages) over 15 min at room temperature with sonication for 40 s in intervals of 10 s with 10 s intermissions. This was followed by incubation with 6 mM 2-Nitro-5-thiocyanatobenzoic acid (NTCB) to cleave proteins after cysteine residues for 30 min at 37°C. Chemically cleaved lysate proteins (100 μg), were subsequently covalently labeled with Sulfo-NHS-biotin (50 μg) (Cat. A8001, ApexBio, Houston, TX) for 1 h. Free biotin molecules were removed via gel filtration. After blocking nonspecific binding sites on the array, an incubation chamber was mounted onto the microarray to permit the loading of the biotinylated, cleaved protein samples. After incubation for 2 h at 20°C, unbound proteins were washed away. The microarray was then incubated for 12 min at 20°C with anti-biotin goat polyclonal antibody (10 μg) (Cat. B3640-1MG, Millipore-Sigma, St. Louis, MO, USA) that was previously labeled with a 50/50 dye mixture of Alexa Fluor 546 dye (Cat. A20002, ThermoFisher, Rockford, IL) and Sulfo-Cyanine3 dye (Cat. 11320, Lumiprobe, Hannover, Germany) for 1 h.

Two 16-bit images from each KAM-2000 microarray were then captured using a ScanArray Reader (Perkin-Elmer). Signal quantification was performed with ImaGene 9.0 from BioDiscovery (El Segundo, CA) with predetermined settings for spot segmentation and background correction. The output of the array consisted of the average normalized net signals (*i.e.,* the average of 2 normalized net signal values of each antibody on the microarray). Standard error and percent standard deviation of 2 separate measurements of globally normalized signal intensity values for each different antibody on the microarray were calculated. Data were determined as percent change from selected controls (% CFC). A positive value corresponds to an increase in signal intensity in response to the treatment, with a value of 100% corresponding to a 2-fold increment in signal intensity. A negative CFC value indicates the degree of reduction in signal intensity from that of the control.

### Quantification and statistical analysis

All quantification and statistical analyses have been described in the corresponding figure legends.

## Supplemental information

Document S1. Figures S1–S10 and Tables S1–S4

Data S1. Antibody microarray phosphorylation, related to Figure 4 Status of P-4423632 treated uninfected THP1-Cells (UP D1) versus THP-1cells Infected with *M. tuberculosis* H37Rv (Ip D1).

Data S2. Antibody microarray phosphorylation, related to Figure 4 Status of P-4423632 treated THP-1 cells Infected with *M. tuberculosis* H37Rv (Ip D1) versus cells infected with *M. tuberculosis* Δ*ptpA* (Ipko1).

Data S3. Antibody microarray phosphorylation, related to Figure 4 Status of uninfected THP1-Cells (UP) versus uninfected THP-1 cells treated with P-4423632 (UP_D1).
