# Supplementary material for: Glycogen synthase kinase 3 inhibition controls Mycobacterium tuberculosis infection
Source: iScience. 2024 Jul 20;27(8):110555. doi: 10.1016/j.isci.2024.110555 (PMC11340618; doi:10.1016/j.isci.2024.110555)
Supplement: Document S1. Figures S1–S10 and Tables S1–S4 [file mmc1.pdf]

iScience, Volume *27*

Supplemental information

Glycogen synthase kinase 3 inhibition controls *Mycobacterium tuberculosis* infection

Sandra Peña-Díaz, Joseph D. Chao, Celine Rens, Hasti Haghdadi, Xingji Zheng, Keegan Flanagan, Mary Ko, Tirosh Shapira, Adrian Richter, Danay Maestre-Batlle, Julio Ortiz Canseco, Maximiliano Gabriel Gutierrez, Khanh Dao Duc, Steven Pelech, and Yossef Av-Gay

Glycogen Synthase Kinase 3 inhibition controls *Mycobacterium tuberculosis* Infection

Sandra Peña-Díaz[1,7], Joseph D. Chao[2,7], Celine Rens[2], Hasti Haghdadi[1], Xingji Zheng[1], Keegan Flanagan[1], Mary Ko[2], Tirosh Shapira[2], Adrian Richter[2,3], Danay Maestre-Batlle[2], Julio Ortiz Canseco[4], Maximiliano Gabriel Gutierrez[4], Khanh Dao Duc[5], Steven Pelech[2,6], and Yossef Av-Gay[1,2,8]*

[1]Department of Microbiology and Immunology, Life Sciences Institute, University of British Columbia, Vancouver, British Columbia, Canada. [2]Department of Medicine, University of British Columbia, Vancouver, British Columbia, Canada. [3]Institut für Pharmazie, Martin-Luther-Universität Halle-Wittenberg, Halle (Saale), Germany. [4] Host-pathogen interactions in tuberculosis Laboratory, The Francis Crick Institute, London, United Kingdom. [5]Department of Mathematics, University of British Columbia, Vancouver, British Columbia, Canada.[6]Kinexus Bioinformatics Corporation, 8755 Ash Street, Vancouver, B.C., Canada.[7]These authors contributed equally.[8]Lead contact: yossi@mail.ubc.ca

Supplemental Information

		WT		ΔptpA	
	**ID compound**	**MIC50 (µM)**	**MIC25 (µM)**	**MIC50 (µM)**	**MIC25 (µM)**
**1**	P-4423632	6.4	2.9	> 25	> 25
**2**	P-0717632	0.4	ND	> 25	> 25
**4**	P-5908342	> 25	1.2	> 25	> 25
**5**	P-5782442	> 25	1.6	> 25	> 25
**6**	P-7657632	1.4	ND	> 25	0.91
**7**	P-3817632	2	0.6	> 25	13.8

Table S1. Comparison of intracellular MIC50 and MIC25 values of GSK3 inhibitors against *M. tuberculosis* WT and *M. tuberculosis* Δ*ptpA*. Related to Figures 1D-F and 3F. The table shows the MIC50 and MIC25 values obtained from the dose-dependency assays testing six selected GSK3 inhibitors against intracellular *M. tuberculosis* H37Rv (WT) and *M. tuberculosis ΔptpA*. The MIC50 and MIC25 values were determined by non-linear regression with the omission of up to two outliers. The MIC25s were not determined (ND) for compounds 2 and 6, as the values were outside the range of the observed x values.

Bacteria	P-4423632	Control (mm)	Control compound (µg)
Acinetobacter baumannii	R	20	Gen (10)
Bacillus subtilis	R	24	Gen (10)
Escherichia coli	R	18	Gen (10)
Enterococcus faecalis	R	ND	ND
Moraxella catarrhalis	R	30	Gen (10)
Pseudomonas aeruginosa	R	21	Gen (10)
Staphylococcus aureus	R	25	Gen (10)
MRSA	R	ND	ND
Staphylococcus epidermidis	R	26	Gen (10)
Salmonella typhimurium	R	20	Gen (10)
Mycobacterium marinum	R	ND	ND
Mycobacterium intracellulare	R	20	Gen (12.5)
Mycobacterium bovis BCG	R	30	Gen (10)
Mycobacterium smegmatis	R (100 µg)	11	BDQ (1.5)
Mycobacterium abscessus	R (100 µg)	8	BDQ (1.5)
Mycobacterium tuberculosis H37Rv auxotroph, mc^2 6206	R (160 µg)	30	BDQ (5)
M. avium avium	R	21	Gen (12.5)

Table S2. Disk assay examining P-4423632 *in vitro* activity against a variety of gram negative, gram positive and mycobacterial strains related to Figure 1. R denotes resistance (no bacterial clearing) up to 25 µg of P-4423632 as the maximum concentration tested unless otherwise indicated; Gen: Gentamycin control; BDQ: Bedaquiline control.

	(M)IC50 (μM)	(M)IC90 (μM)
THP-1	2.31	15.99
hMDM	4.17	NA
In broth	38.81	43.25
Cytotoxicity in THP1	77.80	114.81

Table S3. Minimal inhibitory concentrations of P-4423632 against *Mycobacterium tuberculosis* H37Rv in cell lines and in broth, and inhibitory concentrations (toxicity level) to THP-1 cells. Related to Figure 2.

Primer	Sequence	Source
GAPDH qPCR For primer	GCCTCAAGATCATCAGCAATGC	Av-Gay laboratory, University of British Columbia
GAPDH qPCR Rev primer:	GTGGTCATGAGTCCTTCCACGA	Av-Gay laboratory, University of British Columbia
GSK3α qPCR For primer:	CTTGGCCTACATCCACTCCC	This paper
GSK3α qPCR Rev primer:	CTTTGCACTGCCAAAATCGC	This paper
GSK3β qPCR For primer:	AACTACCAAATGGGCGAGAC	This paper
GSK3β qPCR Rev primer:	TCCGAGCATGAGGAGGAATA	This paper

Table S4. Oligonucleotides used for qPCR analysis of siRNA knock-down of GSK3 in THP-1 cells related to Star Methods Resource Table.

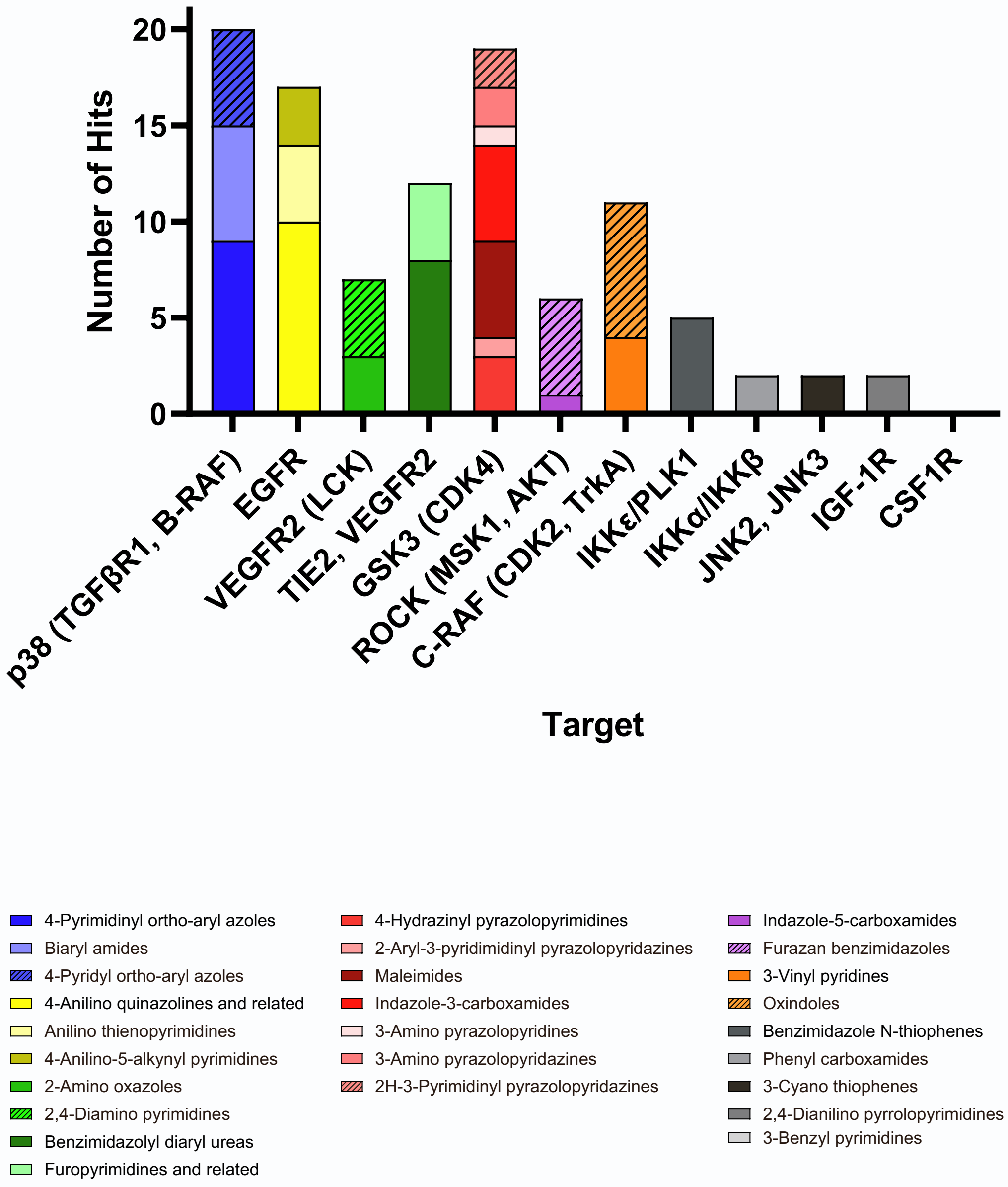

Figure S1: Number of hits per kinase target, related to Figure 1. Of the 313 unique compounds tested in the PKIS/UNC library (Fig 1A), approximately one third (103) displayed at least 20% reduction of intracellular growth of Mtb with an acceptable toxicity in THP-1 cells (viability > 70%). Stacked bars represent the number of active compounds grouped by compound chemotype targeting the same host kinase. Hatched sections represent chemotypes that inhibit more than one kinase (shown in brackets).

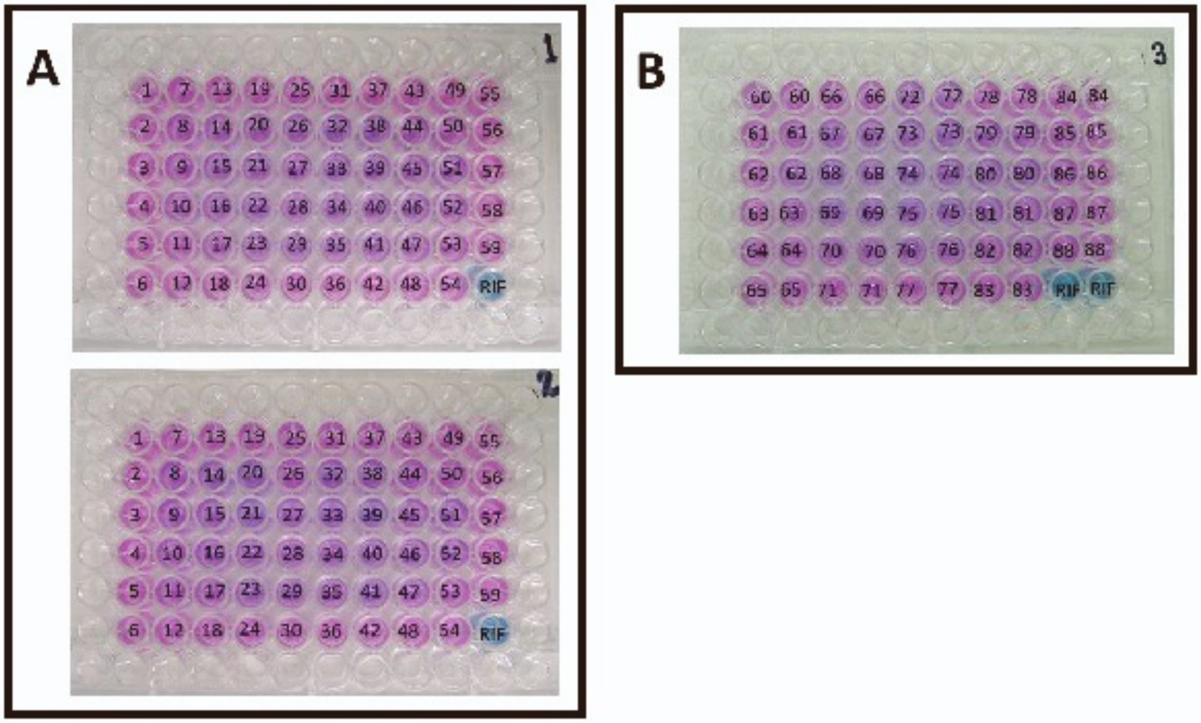

Figure S2: GSK3 inhibitors has limited effect on growth of *M. tuberculosis* in broth, related to Figure 1 and Star Methods Broth Activity analysis. A qualitative analysis of Takeda's GSK3 inhibitor library against Mtb cultured in broth. The images show a resazurin assay of 96 well-plates of *M. tuberculosis* treated with the GSK3 inhibitors or rifampicin (positive control) all at 20 µM concentration. Panel A shows Mtb treated with GSK3 inhibitors #1-59. Panel B shows Mtb treated with GSK3 inhibitors #60-88. Each inhibitor was tested in duplicate. The conversion of resazurin (blue) to resorufin (pink) indicates the presence of live bacteria.

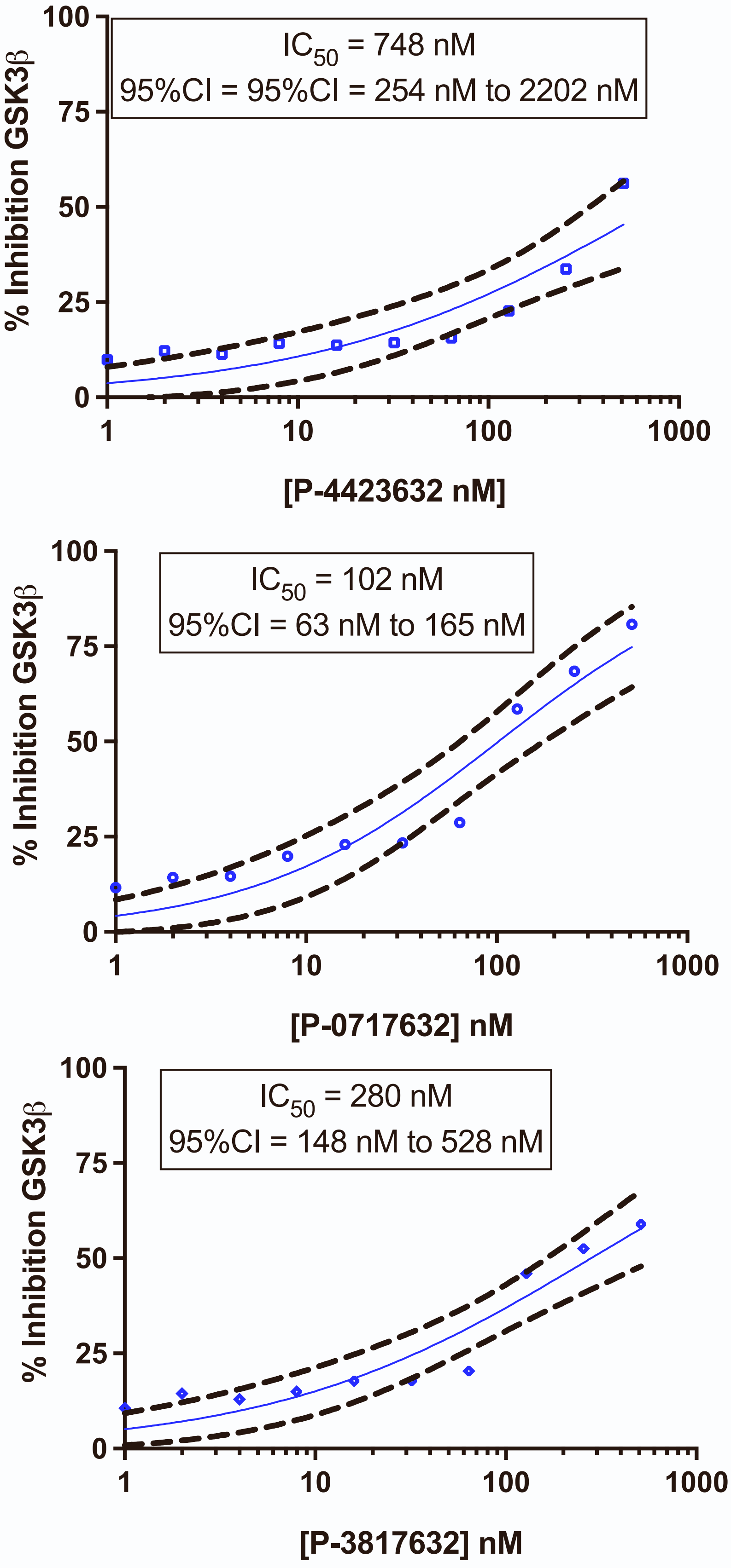

Figure S3: *in vitro* inhibition of GSK3β by selected inhibitors, related to Star Methods: Determination of IC50 of GSK3 inhibitors. GSK3β activity was measured using the ADP-Glo^{TM} Kinase assay and normalized to the negative control (100% kinase activity). Curve was fit using non-linear regression analysis calculated with GraphPad Prism software.

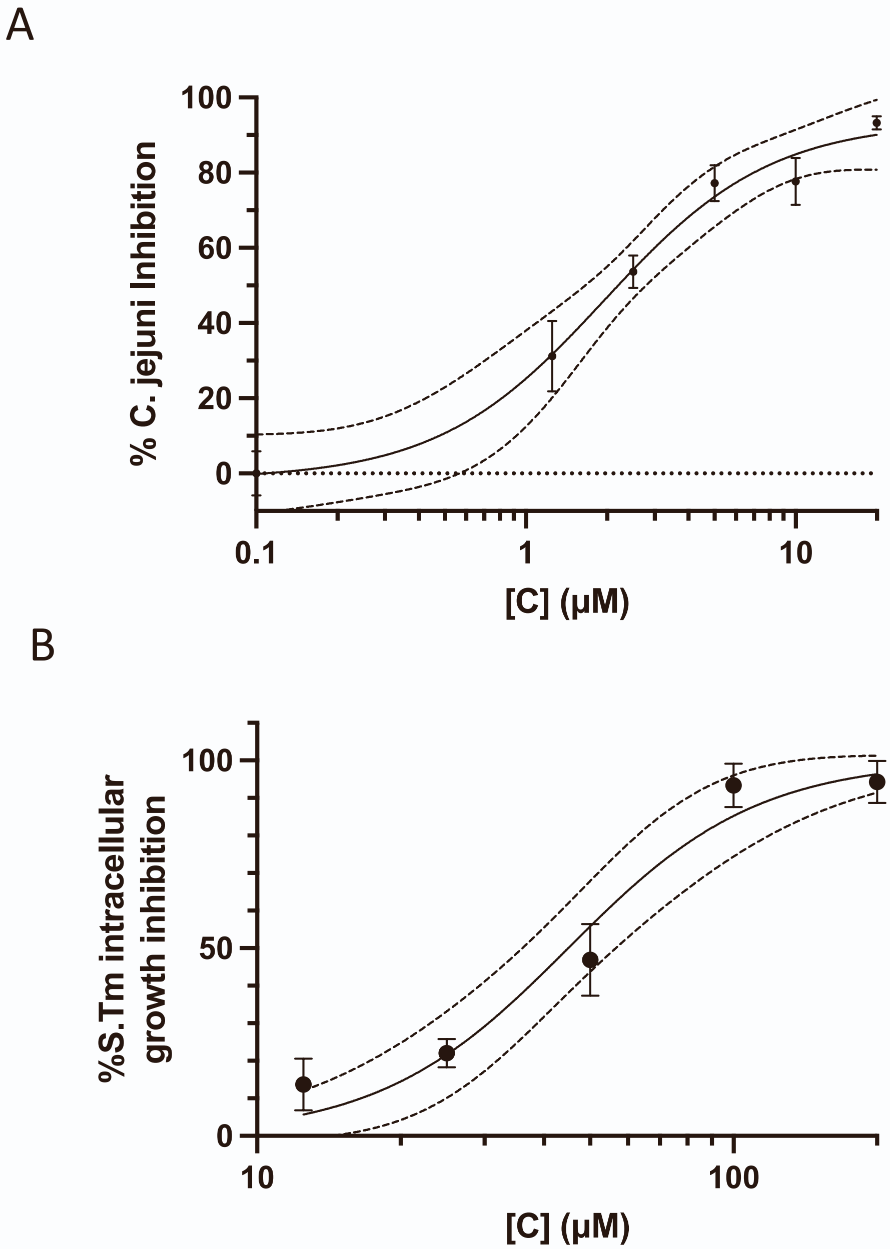

Figure S4: Dose-dependent activity of GSK3β inhibitor P-4423632 on intracellular bacteria. Related to Star Methods describing *C. Jejuni* and *S. enterica* infections. A. Caco-2 cells were infected with *C. jejuni* (MOI 1:500) and treated with two-fold serial dilutions of P-4423632 for 24h. Percent inhibition was calculated by subtracting CFU values as a percentage of the untreated DMSO control (plotted at 0.1 μM) from 100%. Data represents three biological experiments performed in duplicate ± SEM, N = 6. Curve was fit using non-linear regression analysis of log (inhibitor) vs. response (variable slope) calculated with GraphPad Prism 10. **B.** Dose dependent inhibition of *Salmonella enterica* serovar Typhimurium in THP-1 cells (MOI 10:1). Data from three independent experiments. Non-linear regression curve plotted following constraining the range from 0-100% using GraphPad Prism 10.

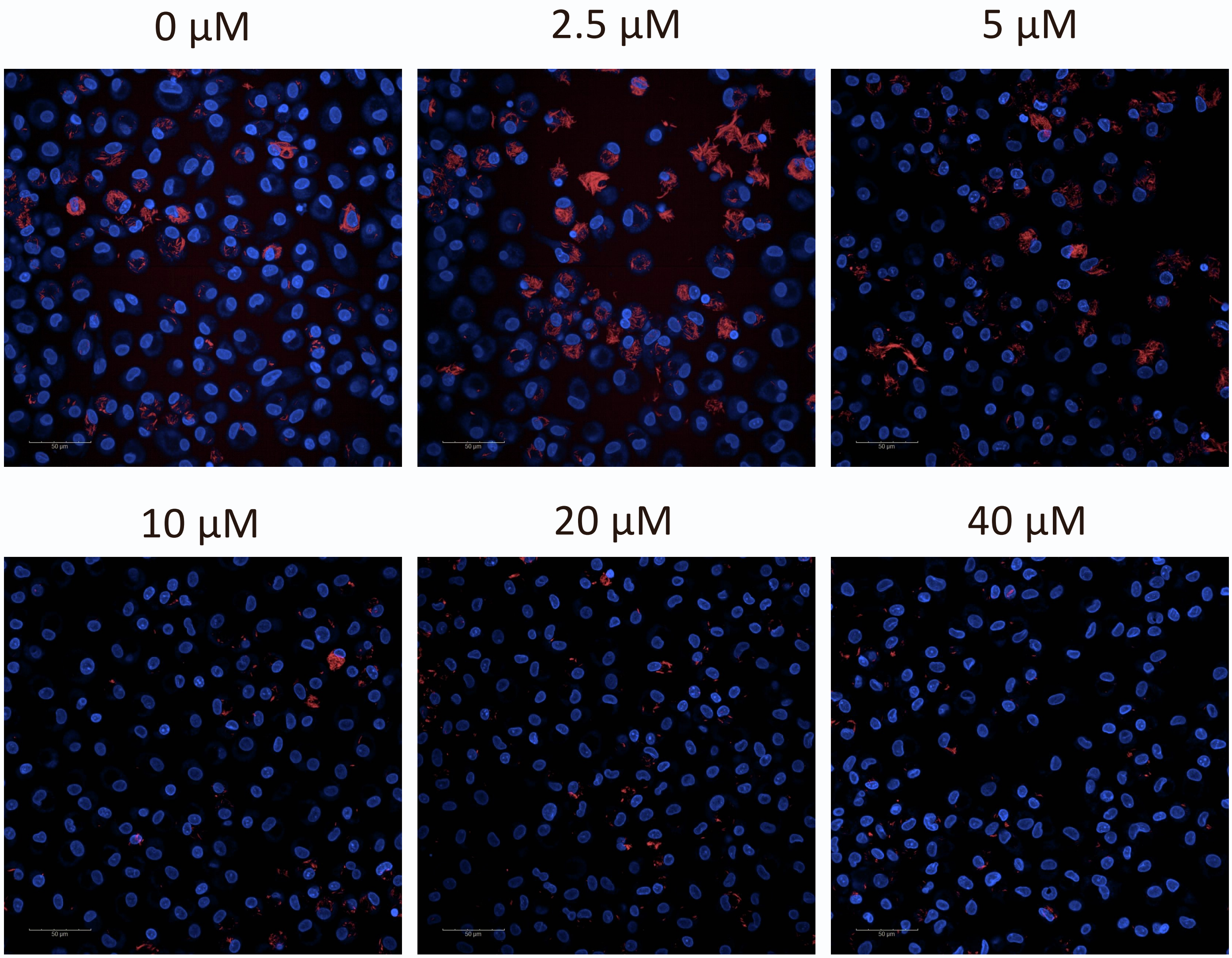

Figure S5: Images from HCS of Mtb-infected hMDMs treated with P-4423632, related to Figure 3A. Representative images taken at 72h post-treatment with two-fold serial dilutions of P-4423632. Images were captured at 40x magnification using the Phoenix Opera HCS system and are a composite of two fluorescent channels: blue = DAPI-stained nuclei of hMDMs, red = Mtb expressing E2-Crimson. Images represent one of 25 fields per well, four wells per compound concentration, of three biological replicates used to calculate the dose-response curve in Fig 3 A.

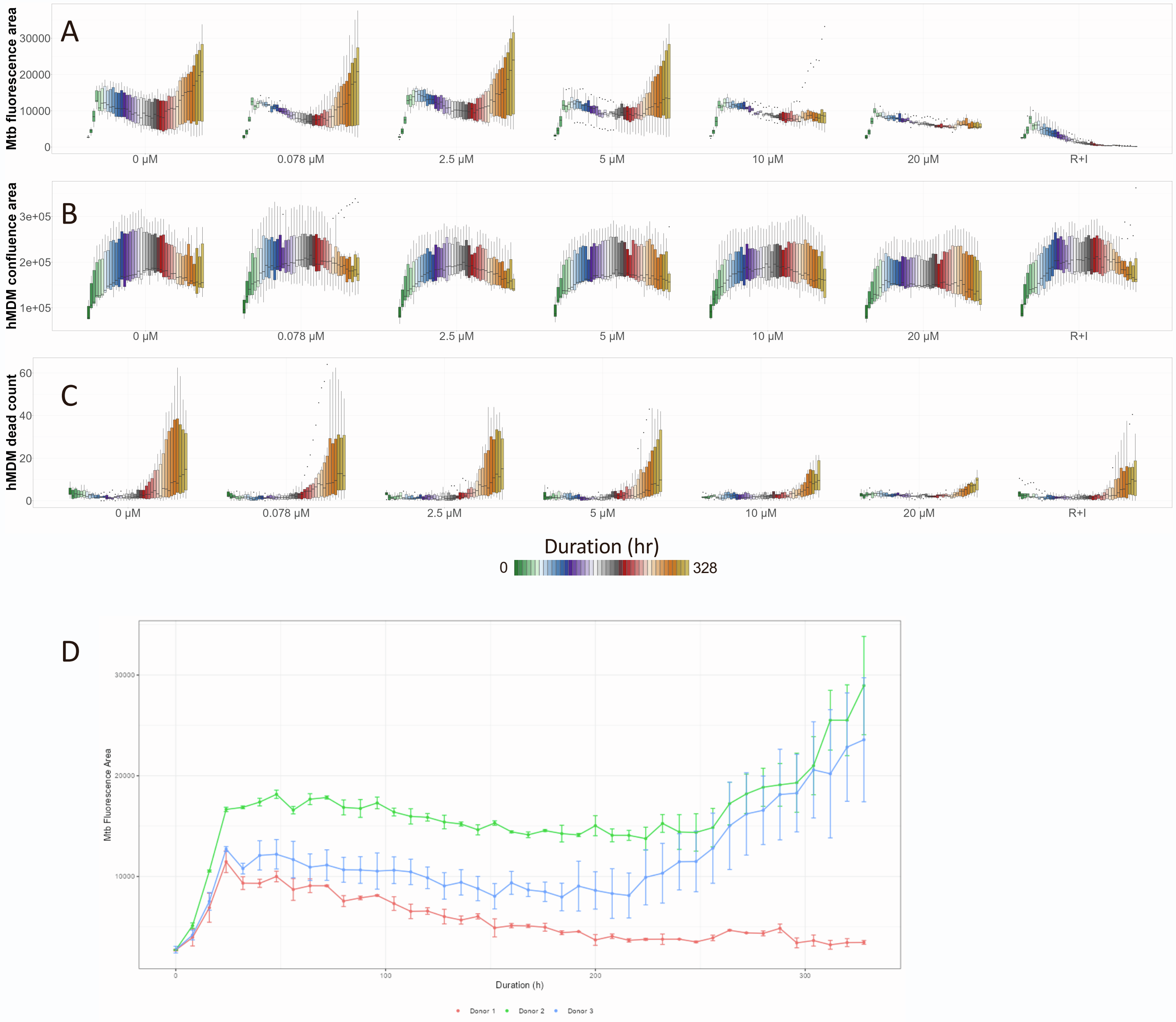

Figure S6: P-4423632 inhibition of Mtb-infected hMDM cells, related to Figure 3A. hMDMs from three independent donors were infected with Mtb expressing GFP and were monitored over the course of 328 h using the Sartorius IncuCyte S3 HCS platform with automated analysis of live cell imaging every 8h **A.** Mtb growth represented by GFP fluorescence area, **B**. Confluence area of hMDMs cells, **C.** hMDMs cell viability (dead count) represented by RFP spot counts of DRAQ7 dye. Infected hMDMs were treated with two-fold serial dilutions of P-4423632 from 20 µM to 0.078 µM (highest four and lowest concentrations shown), as well as 0.1% DMSO vehicle control (0 µM) and 1 µg/mL of Rifampicin and Isoniazid (R+I) positive control. Box and whisker plots represent the median, quartile, and outlier (points) data from three blood donors performed in duplicate (N = 6). **D.** Variability among hMDM donors. Data from A at 0 µM (DMSO control) were plotted as a line graph by donor ± SEM which demonstrated complete control of Mtb by one of the donors and large variability of Mtb control among the donors over the time course of the experiment. *N* = 2.

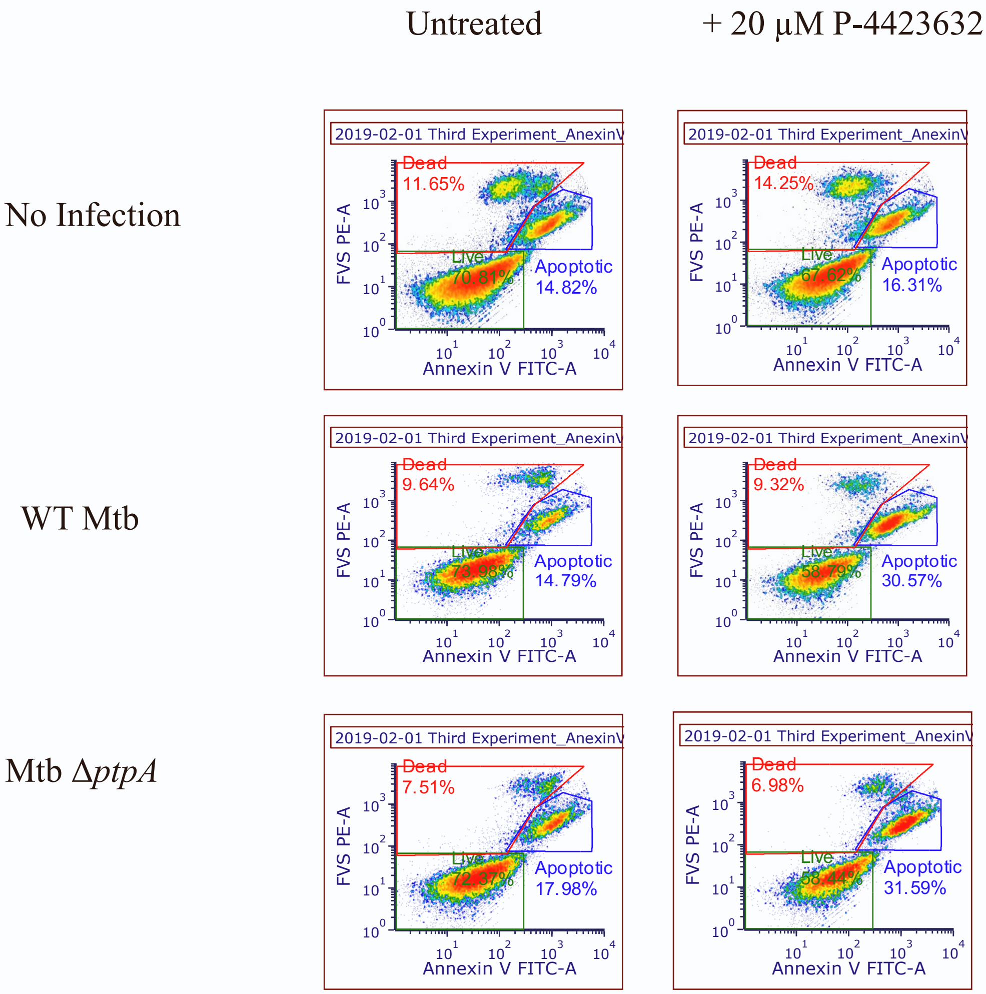

Figure S7 : Flow cytometric dot plots of live, apoptotic, and dead THP-1 cells during infection and treatment with GSK3 inhibitor at 48 hours post-treatment, related to Figure 3C. THP-1 cells were infected with Mtb (WT) or Mtb Δ*ptpA* at an MOI of 5, treated with P-4423632 GSK3 inhibitor at 20 µM, and then harvested at 48 hours post-treatment. Flow cytometric dot plots showing the percentage of live cells (Annexin V- FITC negative and FVS negative cells), dead cells (top right quadrant marked in red) and late-stage apoptotic cells (Annexin V- FITC positive, top left quadrant, marked in blue) at 48 hours post-infection. Each plot is a representative of two samples. Thirty thousand events were measured per sample. 24 and 72 h analysis in duplicates showed similar trends.

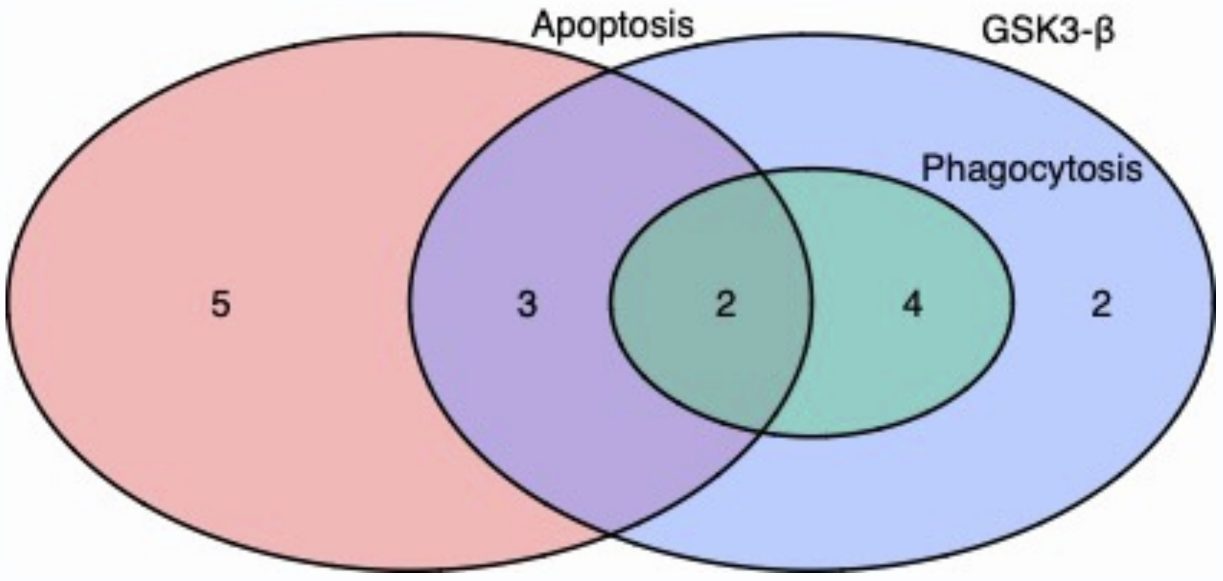

Figure S8:Venn diagram showing the involvement of 16 highly modulated proteins in GSK3, phagocytosis, and apoptosis related pathways, related to Figure 4. GSK3β related pathways are defined as any KEGG pathway which involves GSK3β. Disease specific pathways such as cancer pathways were filtered out. Phagocytosis and apoptosis related pathways were provided directly by KEGG. The 16 proteins were chosen from the 25 proteins with the highest difference in log fold change between infection and infection + treatment as shown in Fig. 4 C. Circle color shows the pathway group: GSK3β = blue, phagocytosis = green, apoptosis = red.

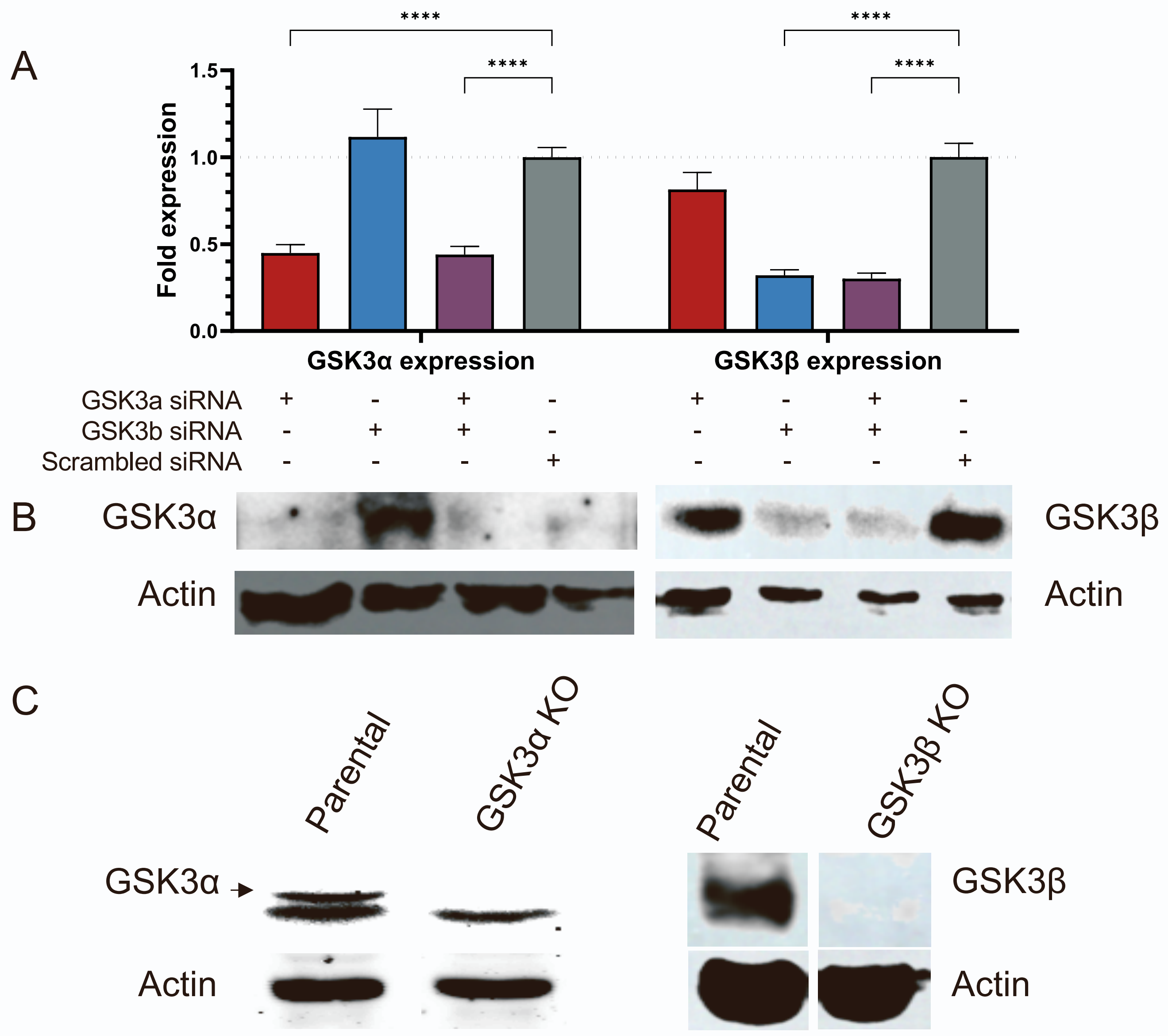

Figure S9: RNA and protein expression levels of GSK3 variants in siRNA knockdown and CRISPR knockout THP-1 cells, related to Figure 2 and Star Methods: siRNA mediated gene silencing of GSK3. A. Confirmation using qPCR of siRNA knockdown of GSK3α and GSK3β RNA levels in THP-1cells following transfection with GSK3α siRNA (blue), GSK3β siRNA (red), and both GSK3α and GSK3β siRNA (purple); siRNA transfection is indicated. Fold expression represents the GSK3 variant expression levels in cells transfected with the indicated GSK3 siRNA compared to transfection with scrambled siRNA control (gray). Data represents the mean + SD of a representative experiment. *N = 4*. Statistics were performed using two-way ANOVA followed by Bonferroni's post hoc test compared to the scrambled siRNA control; *** $p < 0.001$. **B.** Western blot of GSK3α and GSK3β protein levels of THP-1 cells following transfection with the indicated siRNA as in **A.** Actin was used as a loading control. **C.** GSK3α and GSK3β protein levels in their corresponding CRISPR knockout mutants and parental THP-1 cells as measured by Western blot using anti-GSK3α and anti-GSK3β antibodies.

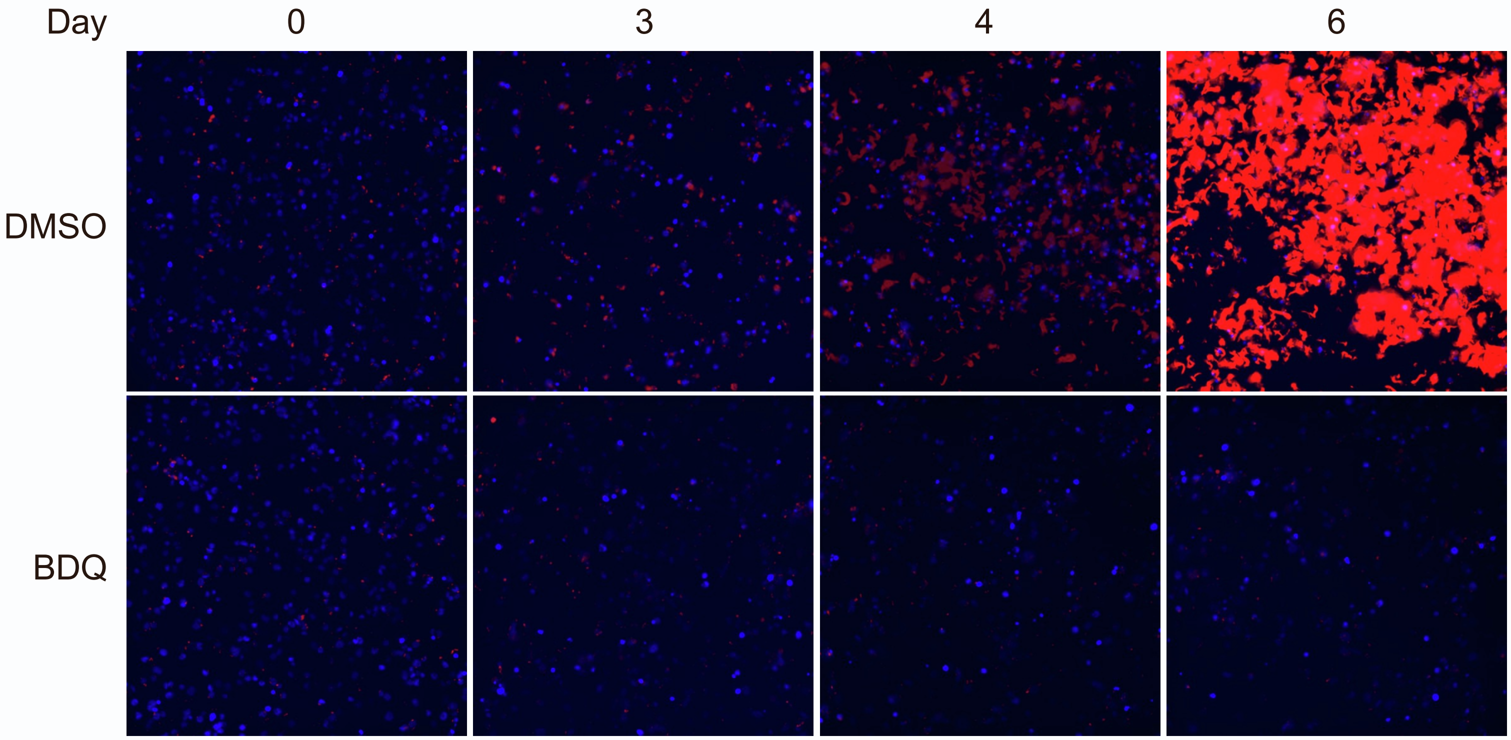

Figure S10: Images from HCS of Mtb-infected THP-1 cells treated with BDQ, related to Star Methods: High-content intracellular screening. Representative time-course images taken over 6 days post-treatment with or without BDQ. Images were captured at 20x magnification using the Molecular Devices ImageXpress Micro 4 HCS system and are a composite of two fluorescent channels: blue = DAPI-stained nuclei of THP-1 cells, red = Mtb expressing RFP. RFP channel was captured with equal exposure times with no adjustment to the RFP images other than false coloring (red).
